# The contribution of stem cell factor and its receptor c-Kit to cancer-induced bone pain

**DOI:** 10.1172/jci.insight.191905

**Published:** 2026-05-05

**Authors:** Kelly F. Contino, Jenna Ollodart, Yang Yu, Sun H. Park, Shunsuke Tsuzuki, Kara Rollins, Tyler M. Heethouse, Joshua Chu, Laiton R. Steele, Takahiro Kimura, Jingyun Lee, Cristina M. Furdui, Lance D. Miller, Fang-Chi Hsu, Yusuke Shiozawa

**Affiliations:** 1 Department of Cancer Biology and Atrium Health Wake Forest Baptist Comprehensive Cancer Center and; 2 Department of Radiation Oncology, Wake Forest University School of Medicine, Winston-Salem, North Carolina, USA.; 3 Department of Urology, The Jikei University School of Medicine, Tokyo, Japan.; 4 Department of PA Studies,; 5 Department of Internal Medicine, Section on Molecular Medicine, and; 6 Department of Biostatistics and Data Science, Wake Forest University School of Medicine, Winston-Salem, North Carolina, USA.

**Keywords:** Neuroscience, Oncology, Pain

## Abstract

Cancer-induced bone pain (CIBP) is among the most common and debilitating symptoms in patients with bone metastasis. Current treatments are somewhat effective but have severe side effects. For the future development of safer CIBP treatments, in this study, we sought to investigate the mechanisms whereby the nerve-cancer interaction controls CIBP. We found that c-Kit, a receptor tyrosine kinase, was activated in the dorsal root ganglia (DRG) sensory neurons of mice with CIBP and that c-Kit’s sole ligand, stem cell factor (SCF), was enhanced in the bone marrow with bone metastasis. When DRGs were treated with SCF or conditioned medium from high SCF-expressing cancer cells, in vitro nerve sprouting was enhanced, and this effect was abolished with c-Kit inhibitors. Mice inoculated intrafemorally with cancer cells that had varying levels of SCF expression developed CIBP and enhanced peripheral nerve sprouting in an SCF-dependent manner. Downstream proteomic analysis revealed that SCF upregulated and activated fibroblast growth factor 1 (FGF1) in DRGs. When FGF1 was knocked down in DRGs, SCF-mediated nerve sprouting was prevented. Taken together, our studies demonstrate the importance of the SCF/c-Kit axis in CIBP and nerve sprouting and identify the SCF/c-Kit/FGF1 pathway as a potential therapeutic target for CIBP.

## Abstract 2

Bone metastatic cancer cells induce bone pain by enhancing sensory nerve sprouting through the stem cell factor (SCF)/c-Kit/fibroblast growth factor (FGF)1 pathway.

## Graphical abstract

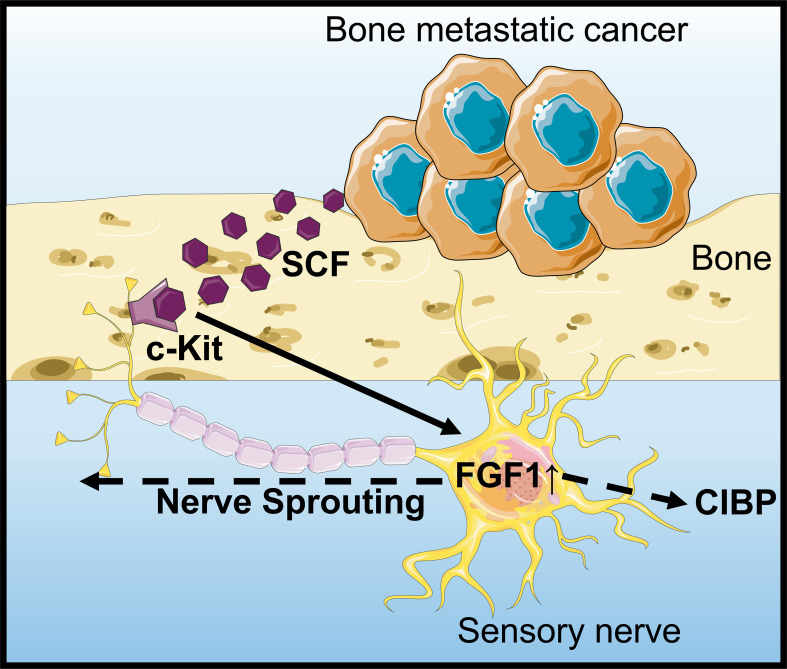

## Introduction

Cancer-induced bone pain (CIBP) represents a complex challenge facing patients with bone metastasis. CIBP affects about 80% of patients with bone metastases and can substantially impair quality of life (1). CIBP is unpredictable in both timing and intensity, adversely affecting day-to-day functioning (2). The complex phenomenon that is CIBP includes background pain, spontaneous pain, and movement-evoked pain, which can present individually or in combination (3); it is distinct from other forms of chronic pain (i.e., inflammatory or neuropathic pain) (4).

Analgesics (e.g., opioids, nonsteroidal anti-inflammatory drugs [NSAIDs]) have been leveraged somewhat effectively for CIBP treatment by reducing inflammation and its associated pain through targeting of the central and peripheral nervous systems; however, these analgesics are associated with severe side effects. NSAIDs cause gastrointestinal ulcers, cardiovascular events, hypertension, and renal failure (5). Opioids lead to constipation, neurotoxicity, respiratory depression, and urinary retention (6). Moreover, opioids are often addictive (6–8). In addition, treatments targeting bone remodeling, such as bisphosphonates, denosumab, and radium-223, can also reduce CIBP; however, these treatments are also associated with severe adverse effects. Specifically, bisphosphonates and denosumab are associated with osteonecrosis of the jaw, and radium-223 leads to fatigue and pancytopenia (3, 9–15). Therefore, therapies more specifically targeting mechanisms of CIBP are needed enhance safety and efficacy for patients.

Bone metastatic cancer and the bone marrow microenvironment (e.g., immune cells) are known to induce CIBP through mechanical stress, acidification, and inflammation (4, 16, 17). Particularly, growth factors, cytokines, and chemokines secreted by cancer and/or the acidic environment surrounding cancer in the bone have been known to stimulate receptors on sensory nerves (4, 16–18). This indicates that CIBP occurs not only during physical contact between cancer cells and sensory nerves, but also from signaling events initiated by factors derived from bone metastatic cancer (4, 16, 19, 20). Moreover, stimulation of sensory nerves by cancer cell–derived secretory factors is known to induce nerve sprouting (21, 22), which is recognized as one of the mechanisms underlying pain, including skeletal pain (23–26). However, the precise molecular mechanisms through which nerve-cancer interactions contribute to CIBP remain elusive.

Receptor tyrosine kinase (RTK) c-Kit is known to be expressed in high levels in the dorsal root ganglia (DRG), a hub of sensory neurons responsible for nociceptive signaling (27–34). These DRG nerve fibers extend to the periphery of the body, including to the bone, where they are susceptible to factors in the bone microenvironment (35, 36). When c-Kit is activated by the binding of its sole ligand stem cell factor (SCF) (37), dimerization, rapid autophosphorylation, and downstream activation of distinct signaling pathways occur (38). It has been demonstrated that SCF/c-Kit signaling stimulates the in vitro sprouting of nerve fibers and survival of DRG sensory neurons without affecting proliferation (32, 33). Moreover, the SCF/c-Kit axis is also involved in pain signaling in several in vivo models. Recombinant murine SCF (rmSCF) induces acute mechanical and thermal hypersensitivity when injected intraperitoneally, intrathecally, or into the left dorsal hind paw in mice (27, 29). Mice that express a mutant, nonfunctional form of c-Kit have significantly lower thermal hypersensitivity, inflammatory pain, and nerve injury–induced pain than mice with wild type c-Kit (28). Furthermore, mice treated with the RTK inhibitor imatinib, targeting c-Kit, have significantly reduced thermal hypersensitivity (29, 39). Most importantly, patients with chronic myeloid leukemia who were treated with imatinib or nilotinib (c-Kit targeting RTK inhibitors) had significantly reduced heat and cold pain sensitivity; however, the effect on tumor size was not considered (39). Although these findings suggest that the SCF/c-Kit axis has been implicated in the development of chronic pain, whether this axis is also crucial for the development of CIBP has yet to be determined.

In this study, we found that (a) c-Kit was activated in the DRGs of mice with bone-inoculated tumor compared with non-tumor-bearing controls; (b) the expression levels of SCF in the bone marrow were enhanced in patients with bone metastasis and bone-tumor bearing mice; (c) when animals were inoculated intrafemorally with high SCF-expressing cancer cells, they had more CIBP behaviors compared with animals inoculated with low SCF-expressing cancer cells; (d) cancer-derived SCF-induced DRG nerve sprouting was blocked with c-Kit inhibitors; (e) SCF treatment upregulated downstream fibroblast growth factor 1 (FGF1), known to be involved in the induction of nerve sprouting (40), in DRG sensory nerves; and (f) when FGF1 was knocked down in DRG neurons, SCF-mediated nerve sprouting was inhibited. Collectively, our current data suggest that cancer-derived SCF induces nerve sprouting and CIBP through activation of its receptor c-Kit and downstream FGF1, and this axis may be a novel therapeutic target for patients with debilitating CIBP.

## Results

### The SCF/c-Kit axis is involved in CIBP.

**Figure 1:**
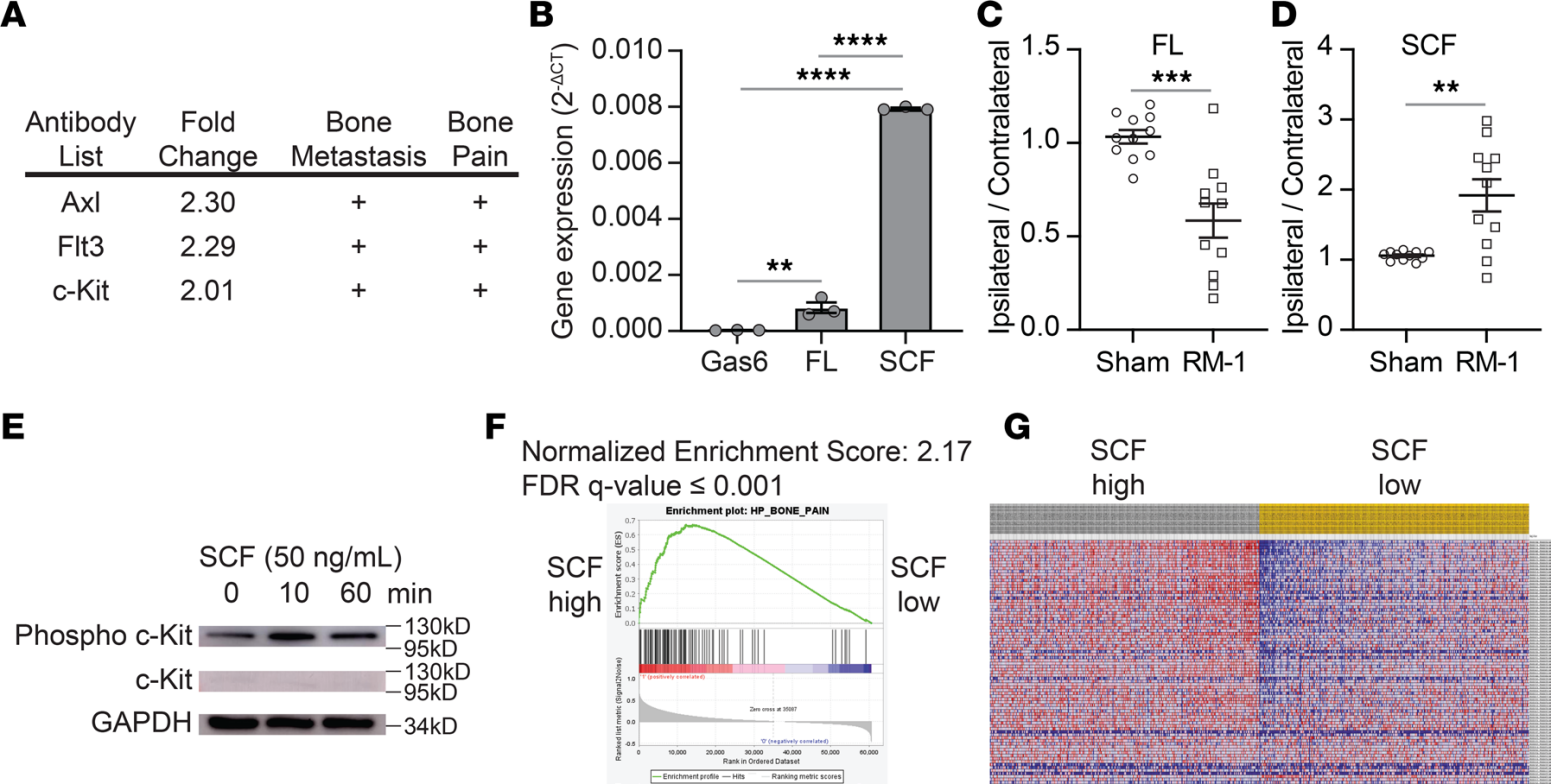
Rationale for studying the SCF/c-Kit axis in CIBP. (**A**) Top 3 candidate pathways identified with (a) antibody-based signaling pathway kinase microarray on DRGs of C57BL/6 mice inoculated intrafemorally with either RM-1 or HBSS (sham) and (b) PubMed literature review. (**B**) RM-1 gene expression of candidate ligands (Gas6, FL, SCF) for receptor tyrosine kinases identified in **D**) (Axl, Flt3, c-Kit), normalized to GAPDH. Mean ± SEM. Student’s *t* test (*P ≤ 0.05; ****P ≤ 0.0001). (**C**) FL and (**D**) SCF protein levels in bone marrow of sham-injected vs. bone RM-1–bearing mice. Mean ± SEM. Student’s *t* test (**P ≤ 0.01, ***P ≤ 0.001). (**E**) Representative Western blot of c-Kit and phospho-c-Kit in DRGs treated with SCF. GAPDH was used as a loading control. (**F** and **G**) GSEA of TCGA prostate cancer dataset (*n* = 554): (**F**) Enrichment plot and (**G**) heatmap of the “bone pain” gene signatures.

To first elucidate the molecular mechanisms behind CIBP, murine prostate cancer RM-1 cells, which we previously validated as a cell line that develops CIBP in mice (41), were inoculated intrafemorally into C57BL/6 mice. In this model, mice exhibited hypersensitivity during the early stages of tumor growth (Supplemental Figure 1A; supplemental material available online with this article; https://doi.org/10.1172/jci.insight.191905DS1); however, this symptom did not correlate with disease progression (Supplemental Figure 1C). Conversely, mice displayed increased spontaneous guarding behavior as the disease advanced (Supplemental Figure 1, B and C). Furthermore, both hypersensitivity and spontaneous guarding behavior were partially alleviated 3 hours after NSAID carprofen treatment (25 mg/kg) based on pharmacokinetic studies as previously described (42, 43) (Supplemental Figure 1, A and B), indicating that this model is suitable for assessing nociception. While mice with intraosseous tumors can develop mechanical hypersensitivity, previous work by Patrick Mantyh’s group showed that skin hypersensitivity does not always correspond to skeletal pain–related behaviors, such as those exhibited during CIBP (44). Therefore, we primarily used spontaneous guarding behavior as a measure of CIBP in subsequent studies. Three weeks after tumor inoculation, DRGs were collected from either bone RM-1–bearing mice or sham-injected mice, and an antibody-based tyrosine phospho-specific protein microarray of these DRGs were performed. Among the 228 tyrosine phosphorylation sites analyzed, 20 tyrosine sites were found to be enhanced in the DRGs from bone RM-1–bearing mice (cutoff: ≥2-fold change, DRGs of bone RM-1–bearing mice/DRGs of sham-injected mice). To further probe these candidate protein phosphorylation pathways, we performed a literature search with terms “bone metastasis” and “bone pain” using PubMed. This search revealed that 5/20 tyrosine phosphorylation sites were involved in “bone metastasis,” and 4/20 tyrosine phosphorylation sites were associated with “bone pain.” Interestingly, only 3/20 of these tyrosine phosphorylation sites were implicated in both “bone metastasis” and “bone pain.” These 3 identified tyrosine phosphorylation sites were Axl, fms-related RTK 3 (Flt3), and c-Kit (Figure 1A), all of which are RTKs. As binding of cancer-derived factors to receptors on sensory neurons is a known mechanism of CIBP (4, 16, 19, 20), we sought to determine the gene expression levels of the 3 identified RTKs’ ligands (Gas6 for Axl, Flt3 ligand [FL] for Flt3, and SCF for c-Kit) in RM-1 cells. RT-qPCR revealed that RM-1 expresses (a) little to no Gas6, (b) moderate levels of FL, and (c) very high levels of SCF (Figure 1B). Thus, the Gas6/Axl pathway was not considered for further evaluation in this study. We then compared the protein levels of FL and SCF between bone marrow obtained from bone RM-1–bearing mice and sham-injected mice by ELISA. The ipsilateral bone marrow from bone RM-1–bearing mice contains both bone marrow cells and a large amount of RM-1 cells, while the contralateral marrow contains only bone marrow cells. The ratio of ipsilateral to contralateral bone marrow in bone RM-1–bearing mice showed lower FL levels compared with the FL ratio in sham mice (Figure 1C), suggesting that RM-1 cells may occupy bone marrow space while lowly expressing FL. On the contrary, the SCF level was higher in the ipsilateral-to-contralateral marrow ratio from bone RM-1–bearing mice compared with the ratio from sham mice (Figure 1D), indicating that RM-1 cells occupying bone marrow space may produce high levels of SCF. As such, the FL/Flt3 axis was not selected for further evaluation in this study. We then validated c-Kit activation mediated by ex vivo rmSCF treatment of DRG tissues by Western blot (Figure 1E). Furthermore, The Cancer Genome Atlas’s (TCGA’s) prostate cancer dataset (*n* = 554) was stratified into high and low SCF expression groups and then analyzed using a GSEA of 82 genes comprising the “bone pain” gene signature (45, 46). High SCF expression in patients with prostate cancer positively correlated with the “bone pain” gene signature (false discovery rate [FDR] *q* ≤ 0.001, normalized enrichment score: 2.17) (Figure 1, F and G), suggesting that high SCF expression in prostate cancer is associated with these patients’ susceptibility to bone pain, although (a) these results could not be corroborated, as patient pain scores were not available and (b) a prospective study would be necessary in order to evaluate pain perception and control for other variables, including age, cancer stage, patients’ prognosis, patients’ comorbidities, and patients’ socioeconomic status, etc. Based on these findings, we pursued the SCF/c-Kit axis for further study.

### Peptidergic murine DRGs have higher levels of c-Kit expression than nonpeptidergic murine DRGs.

**Figure 2:**
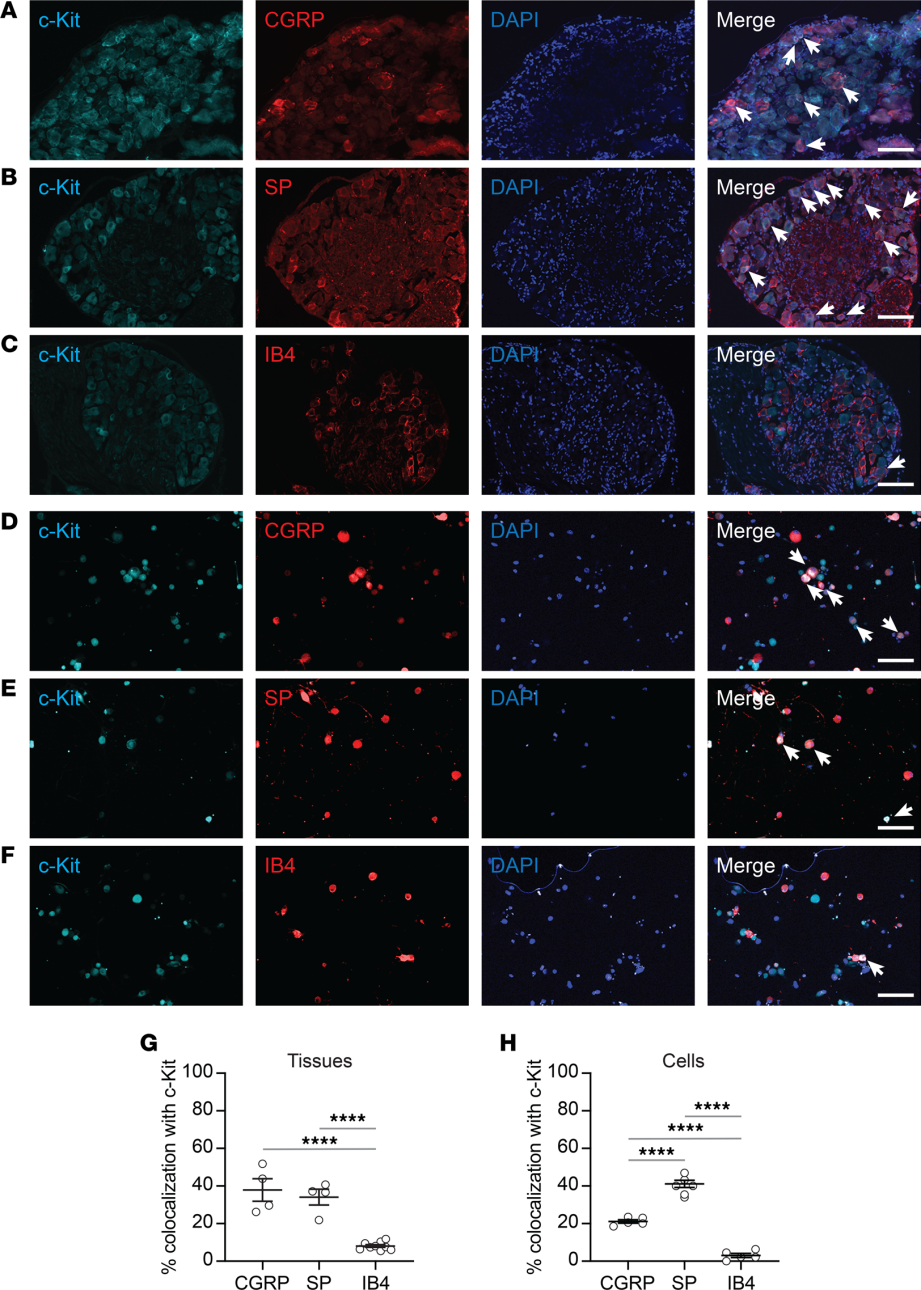
c-Kit is expressed in peptidergic murine DRGs. Representative IF images of colocalization between c-Kit and (**A**) CGRP, (**B**) SP, or (**C**) IB4 in L2–L5 DRG tissue of naive C57BL/6 mice. Original magnification, ×10. Scale bar: 100 μm. Representative IF images of colocalization between c-Kit and (**E**) CGRP, (**F**) SP, or (**G**) IB4 in primary DRG cells obtained from naive C57BL/6 mice. Original magnification, ×10. Scale bar: 100 μm. Arrows indicate colocalization. (**G** and **H**) Quantification of **A**–**C** (**G**) and **D**–**F** (**H**). Mean ± SEM. One-way ANOVA with Tukey’s multiple comparisons (****P ≤ 0.0001).

Prior to determining the specific mechanisms whereby SCF derived from cancer cells may contribute to CIBP development through binding to c-Kit on sensory neurons, we first validated whether naive murine DRG sensory neurons express c-Kit using immunofluorescence (IF). As expected, c-Kit was detected in the DRGs and colocalized with calcitonin gene–related peptide–positive (CGRP-positive) cells in 37.8% ± 6.0% of the c-Kit–positive cells; additionally, substance P–positive (SP-positive) DRGs were detected in 34.1% ± 4.2% of the c-Kit–positive cells, although isolectin B4–positive (IB4-positive) DRGs only colocalized with c-Kit in 8.1% ± 0.7% of the c-Kit–positive cells (Figure 2, A–C and G), suggesting that c-Kit expression colocalizes more in peptidergic neurons compared with nonpeptidergic neurons, as previously demonstrated (47). Consistently, similar findings were upheld in murine primary DRG cell culture model that we previously established (22) (Figure 2, D–F and H).

### Bone metastatic cancer cells express and secrete SCF but do not express c-Kit.

**Figure 3:**
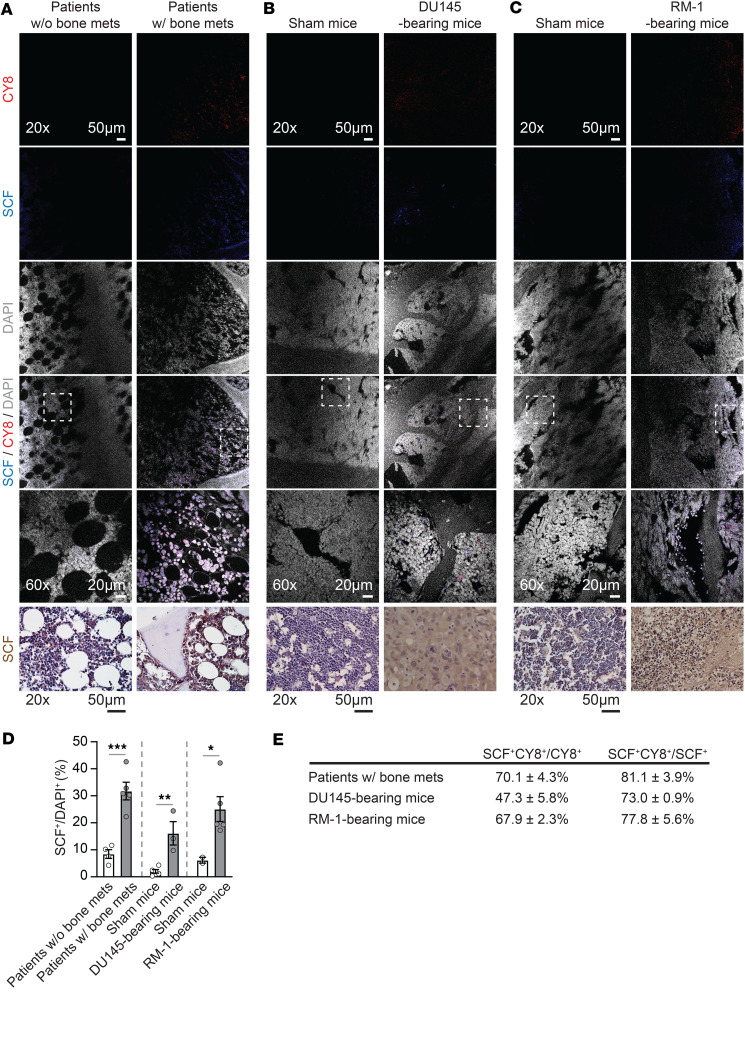
SCF is enriched in bone metastatic cancer cells in the bone marrow. Representative IF images of colocalization between cytokeratin-8 (CY8) and SCF and IHC of SCF in (**A**) bone marrow autopsy samples from patients with prostate cancer who died from other causes (patients without bone mets, *n* = 4) or bone metastases (patients with bone mets, *n* = 5); (**B**) bone marrow of immunodeficient mice inoculated intrafemorally with HBSS (sham mice) and DU145 (DU145-bearing mice) at 54 days posttumor inoculation; and (**C**) bone marrow of immunocompetent mice inoculated intrafemorally with HBSS (sham mice) and RM-1 (RM-1–bearing mice) at 21 days posttumor inoculation. DAPI was used for nuclear staining. Original magnification, ×20; scale bar: 50 μm for IHC images (first 4 rows and last row). Original magnification, ×60; scale bar: 20 μm for IF images (fifth row). (**D**) Quantification of SCF positive cells out of total bone marrow cells of (**A**–**C**). Mean ± SEM. Student’s *t* test (*P ≤ 0.05, **P ≤ 0.01, ***P ≤ 0.001). (**E**) Percentage of SCF expressing CY8 positive cancer cells (SCF^+^CY8^+^/CY8^+^) and CY8 expressing SCF positive cells (SCF^+^CY8^+^/SCF^+^) of (**A**–**C**). Mean ± SEM.

Next, we sought to validate and determine the source of SCF in the bone metastatic microenvironment. Colocalization between SCF and cancer marker cytokeratin-8 in the bone marrow was assessed by IF (Figure 3, A–C, and Supplemental Figure 2) with the antibodies specific to SCF and cytokeratin-8 (Supplemental Figure 3). Higher levels of SCF were observed in the bone marrow of autopsy samples obtained from patients with prostate cancer with bone metastases compared with those without bone metastases (Figure 3, A and D). In the bone marrow of patients with prostate cancer with bone metastases, about 70% of cancer cells (cytokeratin-8–positive cells) expressed SCF and about 80% of SCF-expressing cells were cancer cells (Figure 3, A and E). These data suggest that (a) the bone marrow–containing cancer cells showed elevated SCF levels and (b) a majority of SCF-expressing cells in the bone marrow were bone metastatic cancer cells, although other cells that exist in the marrow may also contribute to increased SCF levels. Similar patterns were observed in the bone marrow of mice inoculated intrafemorally with either DU145 cells or RM-1 cells (Figure 3, B–E), consistent with Figure 1D.

**Figure 4:**
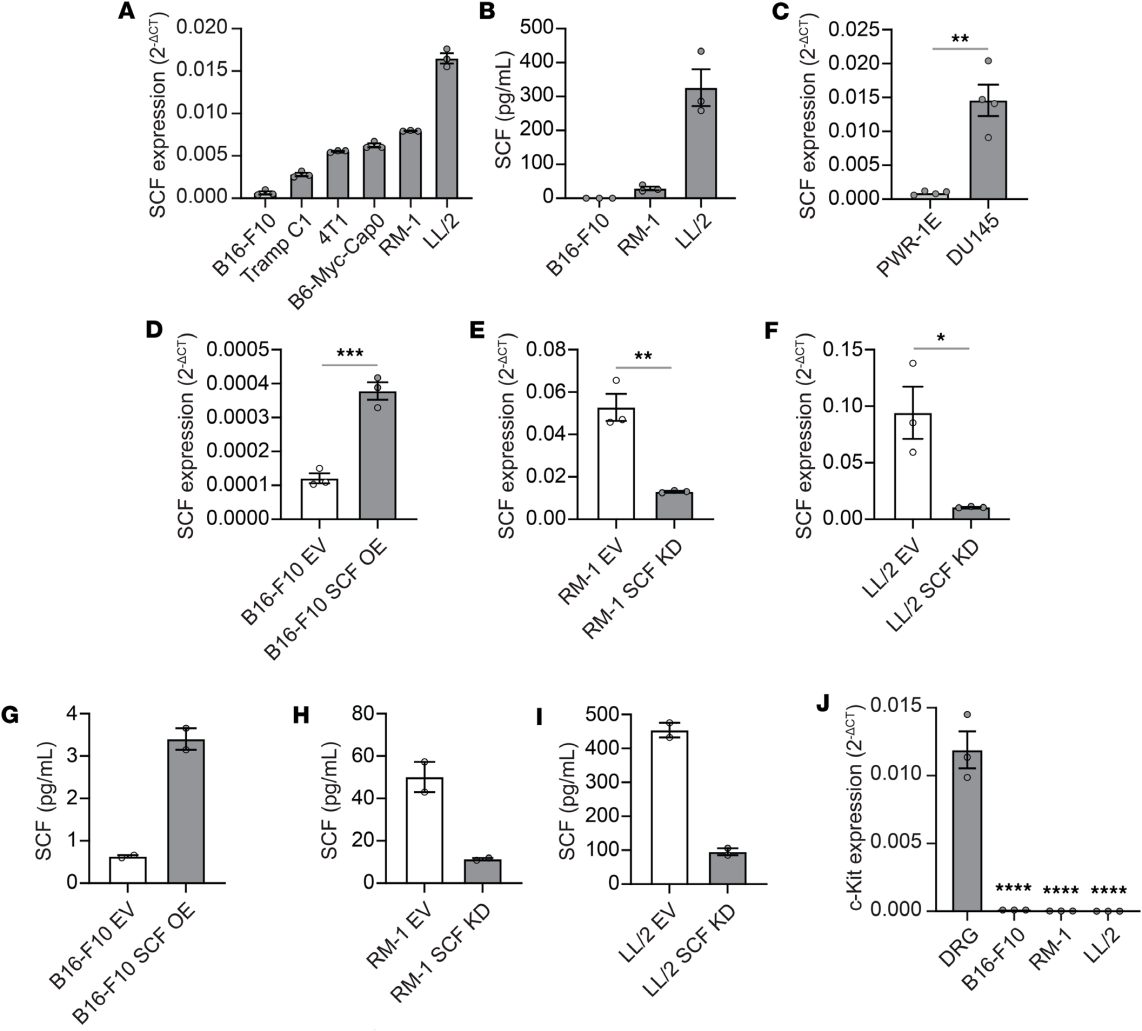
SCF production and c-Kit expression in murine cancer cell lines. (**A**) SCF expression in murine cancer cells, normalized to GAPDH. (**B**) Levels of secreted SCF from B16-F10, RM-1, or LL/2 cells, measured by ELISA. (**C**) SCF expression in human PWR-1E and DU145, normalized to GAPDH. Mean ± SEM. Student’s *t* test (**P ≤ 0.01). (**D**–**F**) SCF expression in B16-F10, RM-1, or LL/2 cells in which SCF levels were genetically altered, normalized to GAPDH: (**D**) control B16-F10 (B16-F10 empty vector [EV]) vs. SCF-overexpressing B16-F10 (B16-F10 SCF OE); (**E**) control RM-1 (RM-1 EV) vs. SCF-downregulated RM-1 (RM-1 SCF knockdown [KD]); or (**F**) control LL/2 (LL/2 EV) vs. SCF-downregulated LL/2 (LL/2 SCF KD). Mean ± SEM. Student’s *t* test (*P ≤ 0.05, **P ≤ 0.01, ***P ≤ 0.001). (**G**–**I**) Levels of secreted SCF from (**G**) B16-F10 EV vs. B16-F10 SCF OE; (**H**) RM-1 EV vs. RM-1 SCF KD; or (**I**) LL/2 EV vs. LL/2 SCF KD, measured by ELISA. Mean ± SEM. (**J**) c-Kit expression in murine DRGs and murine cancer cells, normalized to GAPDH. Mean ± SEM. One-way ANOVA with Tukey’s multiple comparisons (****P ≤ 0.0001).

**Figure 5:**
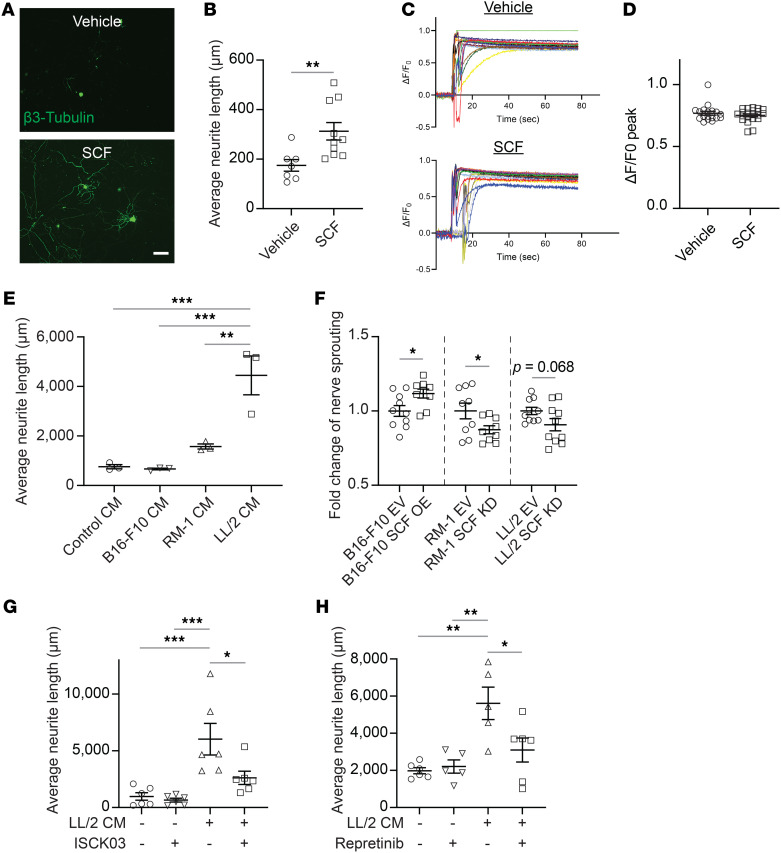
The SCF/c-Kit axis is responsible for in vitro nerve sprouting. (**A**) Representative IF images of β3-tubulin–positive (a pan neuronal marker) neurite outgrowth of murine primary DRG cells treated with SCF. Original magnification, ×10. Scale bar: 100 μm. (**B**) Quantification of neurite outgrowth in **A**. Mean ± SEM. Student’s *t* test (**P ≤ 0.01). (**C**) Representative single-cell traces showing calcium changes in murine primary DRG cells incubated with Fura-2 in response to treatment with vehicle or SCF (24 hours) prior to depolarization with high KCl (50 mM). Fluorescent signals are scaled as ΔF/F_0_ and Fura-2 fluorescent signals are presented as 408/510 nm. (**D**) Quantification of peak calcium influx by change in fluorescent intensity shown in **C**. Mean ± SEM. Student’s *t* test (not significant). (**E**) Neurite outgrowth of murine primary DRG cells treated with serum-free growth medium (control conditioned medium [CM]) or CM obtained from B16-F10, RM-1, or LL/2 cells. Mean ± SEM. One-way ANOVA with Tukey’s multiple comparisons (**P ≤ 0.01, ***P ≤ 0.001). (**F**) Neurite outgrowth of murine primary DRG cells treated with CM obtained from SCF-overexpressing B16-F10 (B16-F10 SCF OE) and SCF-downregulated RM-1 (RM-1 SCF knockdown [KD] or SCF-downregulated LL/2 [LL/2 SCF KD]) vs. CM obtained from respective control cells that were transfected with empty vector (EV). Mean ± SEM. Student’s *t* test (*P ≤ 0.05). (**G** and **H**) Neurite outgrowth of murine primary DRG cells treated with or without LL/2 cell CM in the presence or absence of c-Kit inhibitors, (**G**) ISCK03 and (**H**) repretinib.

**Figure 6:**
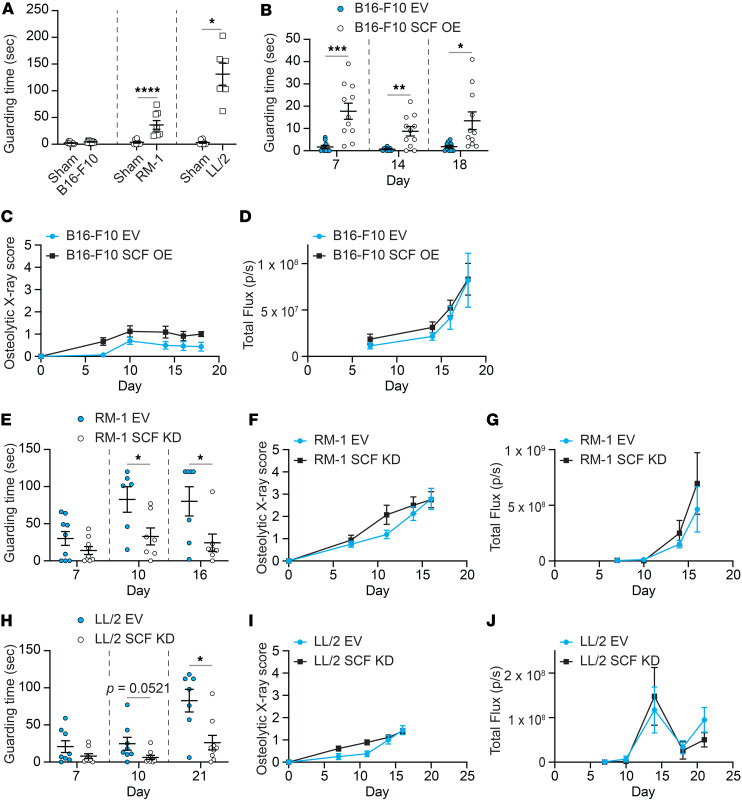
Cancer-derived SCF induces CIBP in vivo but does not alter bone remodeling or tumor growth. (**A**) Guarding behavior quantification of mice inoculated intrafemorally with HBSS (sham), B16-F10, RM-1, or LL/2 cells (day 16). Mean ± SEM. Student’s *t* test (*P ≤ 0.05, ****P ≤ 0.0001). (**B**, **E**, and **H**) Guarding behavior quantification of mice inoculated intrafemorally with luciferase-expressing B16-F10, RM-1, and LL/2 cells in which SCF levels were genetically altered: (**B**) control B16-F10 (B16-F10 empty vector [EV]) vs. SCF-overexpressing B16-F10 (B16-F10 SCF OE); (**E**) control RM-1 (RM-1 EV) vs. SCF-downregulated RM-1 (RM-1 SCF knockdown [KD]); or (**H**) control LL/2 (LL/2 EV) vs. SCF-downregulated LL/2 (LL/2 SCF KD). Mean ± SEM. Student’s *t* test (*P ≤ 0.05, **P ≤ 0.01, ***P ≤ 0.001). (**C**, **F**, and **I**) Osteolytic bone remodeling x-ray scoring and (**D**, **G**, and **J**) tumor growth measured by bioluminescence imaging of the same cohort of mice shown in **B**, **E**, and **H**. Mean ± SEM. Mixed-effects model (not significant).

**Figure 7:**
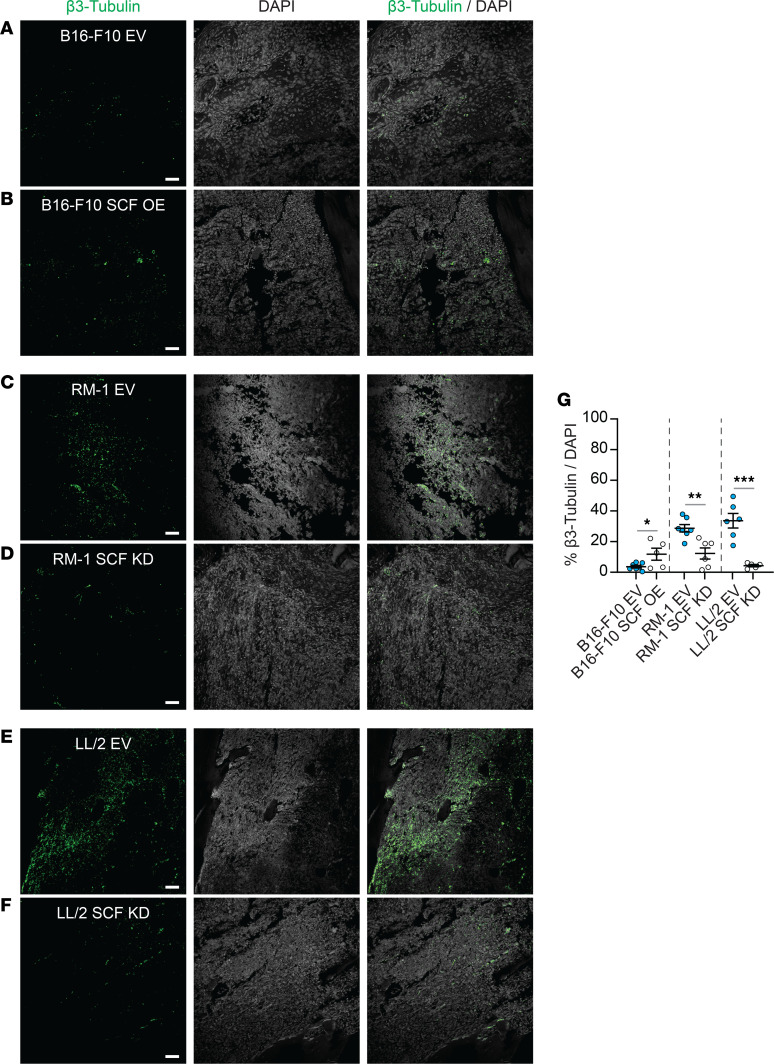
Cancer-derived SCF induces nerve sprouting in vivo. Representative IF images of β3-tubulin–positive (a pan neuronal marker) nerves in ipsilateral femurs of the mice shown in Figure 6, B–J. Original magnification, ×10; scale bar: 100 μm. (**A**) Control B16-F10 (B16-F10 empty vector [EV]) vs. (**B**) SCF-overexpressing B16-F10 (B16-F10 SCF OE); (**C**) control RM-1 (RM-1 EV) vs. (**D**) SCF-downregulated RM-1 (RM-1 SCF knockdown [KD]); and (**E**) control LL/2 (LL/2 EV) vs. (**F**) SCF-downregulated LL/2 (LL/2 SCF KD). (**G**) Quantification of **A**–**F**. Mean ± SEM. Student’s *t* test (*P ≤ 0.05, **P ≤ 0.01, ***P ≤ 0.001).

**Figure 8:**
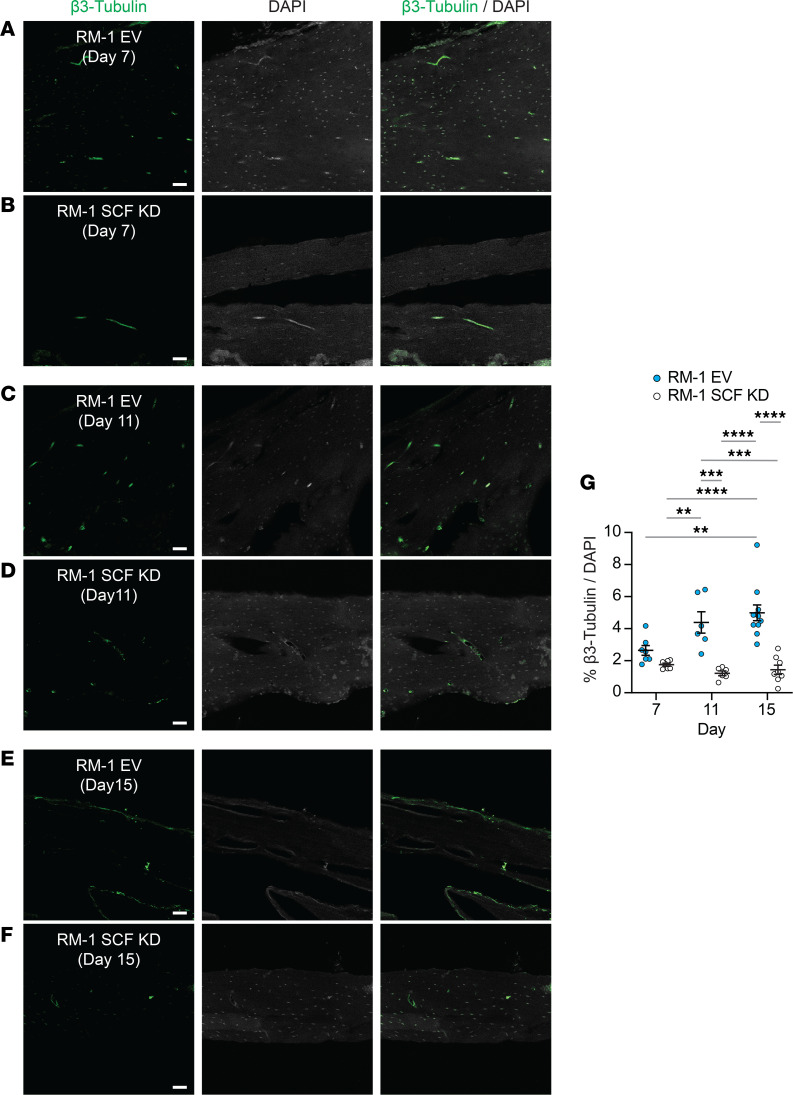
Nerve sprouting in the bone induced by tumors with high SCF expression is associated with disease progression. Representative IF images of β3-tubulin–positive (a pan neuronal marker) nerves in ipsilateral femurs of the mice inoculated with (**A**, **C**, and **E**) control RM-1 (RM-1 EV) vs. (**B**, **D**, and **F**) SCF-downregulated RM-1 (RM-1 SCF knockdown [KD]) at days 7, 11, and 15 posttumor inoculation, respectively. Original magnification, ×10; scale bar: 100 μm. (**G**) Quantification of **A**–**F**. Mean ± SEM. Two-way ANOVA with Tukey’s multiple comparisons (**P ≤ 0.01, ***P ≤ 0.001, ****P ≤ 0.0001).

We then tested several murine cancer cell line levels of SCF in order to explore the role of the SCF/c-Kit axis in CIBP in the syngeneic condition. We found that murine lung cancer cell lines LL/2 and RM-1 expressed higher levels of SCF mRNA compared with other cells (Figure 4A). Very low SCF expression was observed in murine melanoma cell line B16-F10 (Figure 4A). LL/2 and RM-1 cells secreted SCF into their conditioned media (CM), but no SCF secretion was observed from B16-F10 cells (Figure 4B). Thus, LL/2 and RM-1 cells were used as SCF-positive cell lines, and B16-F10 cells were used as a SCF-negative cell line for further study. As Figure 3B, DU145 cells expressed higher levels of SCF mRNA, whereas normal human prostate epithelial PWR-1E cells expressed little to no SCF mRNA (Figure 4C). Next, using a lentiviral vector SCF expression was downregulated in LL/2 and RM-1 cells and upregulated in B16-F10 cells. Knockdown (KD) and overexpression was validated using RT-qPCR and ELISA (Figure 4, D–I). These cells were used for further studies (shown in Figure 5F; Figure 6, B–J; Figure 7; and Figure 8). Importantly, these cells expressed only very low levels of c-Kit, compared with murine DRG (Figure 4J).

### The SCF/c-Kit axis is responsible for in vitro nerve sprouting.

Next, we sought to determine whether SCF could induce nerve sprouting, a mechanism of pain (23–26), through c-Kit using a murine primary DRG culture model (22). rmSCF treatment significantly enhanced nerve sprouting compared with vehicle (Figure 5, A and B). In an effort to explore alternate methods of nociception mediated by SCF, we performed calcium imaging (48–50). Our results demonstrate that DRGs treated with/without SCF do not significantly alter calcium influx (Figure 5, C and D). Therefore, nerve sprouting assays were used in this study to further determine the functional effects of the SCF/c-Kit axis on sensory neurons. Given our findings that both LL/2 and RM-1 cells secrete SCF while B16-F10 cells do not, we then wanted to see if their CM could induce nerve sprouting in vitro. While no effects on cellular proliferation were observed (data not shown), CM from LL/2 and RM-1 cells induced DRG nerve sprouting, whereas CM from B16-F10 cells did not (Figure 5E). Although other factors by these cancer cells may contribute to this phenomenon, these data suggest that cancer CM induced DRG nerve sprouting in an SCF-dependent manner, with greater SCF levels leading to increased sprouting. To address this hypothesis, we repeated nerve sprouting assays using cancer cells with modified SCF levels established in Figure 4, D–I. B16-F10 cell–SCF-overexpressing CM induced increased nerve spouting compared with B16-F10 cell empty vector (EV) CM (Figure 5F), even though these cells expressed relatively low SCF levels (Figure 4, D and G). Conversely, when SCF levels were downregulated in LL/2 and RM-1 cells, neurite outgrowth was reduced, although it did not reach statistical significance in DRGs treated with LL/2 cell CM (Figure 5F). Furthermore, 2 c-Kit inhibitors were able to significantly reduce DRG nerve sprouting mediated by high SCF–secreting LL/2 cell CM (Figure 5, G and H). These data suggest that the SCF/c-Kit axis plays an important role in nerve sprouting in vitro.

### Cancer-derived SCF induces CIBP, but not tumor growth or bone remodeling, by enhancing nerve sprouting.

To test the effects of the SCF/c-Kit axis on CIBP development in vivo, parental B16-F10, RM-1, and LL/2 cells were inoculated intrafemorally into mice, and CIBP was evaluated through spontaneous guarding, a measure of ongoing pain-like behaviors, including those associated with skeletal pain (51). Our results demonstrated that no CIBP was observed in B16-F10 cell–bearing mice, and guarding behavior was similar to that of sham-inoculated mice (Figure 6A). Conversely, both RM-1 and LL/2 cell–bearing mice exhibited significantly greater CIBP compared with their respective sham-inoculated mice (Figure 6A). Next, SCF-overexpressing B16-F10 or SCF-downregulated RM-1 and LL/2 cells were inoculated intrafemorally into C57BL/6 mice. Notably, mice with SCF-overexpressing B16-F10 cells had significantly enhanced CIBP, compared with those with EV-transfected B16-F10 cells (Figure 6B). Conversely, mice with SCF-downregulated RM-1 (Figure 6E) and LL/2 (Figure 6H) cells showed significantly reduced CIBP compared with their respective EV-transfected controls. The manipulation (either overexpression or downregulation) of SCF did not alter bone remodeling in the femur (Figure 6, C, F, and I) or tumor growth (Figure 6, D, G, and J) between SCF-manipulated cell-bearing mice and their respective EV-transduced cell-bearing mice. We then examined nerve fiber sprouting, a known mechanism of CIBP (23–26), in the ipsilateral bone and found that mice inoculated with SCF-overexpressing B16-F10 cells demonstrated enhanced sprouting compared with those with EV-transfected B16-F10 cells (Figure 7, A, B, and G), while mice inoculated with SCF-downregulated RM-1 and LL/2 cells exhibited reduced nerve fiber sprouting compared with their respective EV-transfected controls (Figure 7, C–G). Moreover, enhanced nerve sprouting induced by tumors with high SCF expression was associated with disease progression (Figure 8). These results suggest that SCF derived from cancer cells is integral to CIBP development, presumably by SCF binding to those peripheral nerve fibers that extend from the DRG to the bone.

### SCF enhances and activates downstream FGF1 and induces nerve sprouting through FGF1.

**Figure 9:**
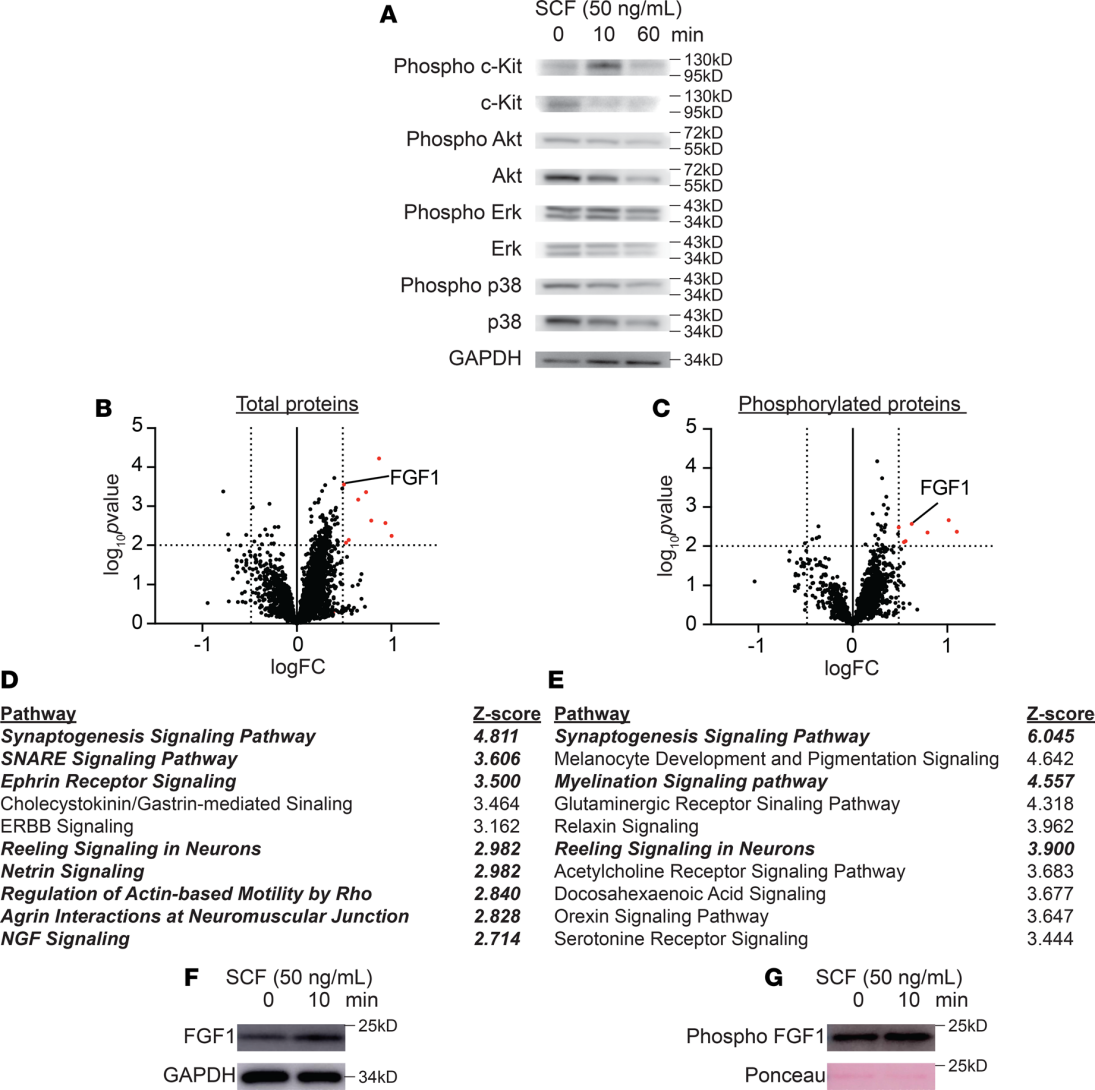
FGF1 is downstream of the SCF/c-Kit axis in murine DRGs. (**A**) Representative Western blot of c-Kit Akt, Erk, and p38 and phosphorylated counterparts in DRGs treated with SCF. GAPDH was used as a loading control. (**B** and **C**) Volcano plot of mass spectrometry of (**B**) total proteins and (**C**) phosphorylated proteins in DRGs treated with SCF. Cutoff: –log_10_*p*value = 2 and log fold change (FC) = 0.5. Red dots indicate the proteins enhanced (9 proteins) or phosphorylated (7 proteins) with SCF. (**D** and **E**) Top canonical pathways from Ingenuity Pathway Analysis of **B** and **C**. (**F** and **G**) Representative Western blot of (**F**) FGF1 and (**G**) phospho-FGF1 in DRGs treated with SCF. Ponceau or GAPDH were used as loading controls.

**Figure 10:**
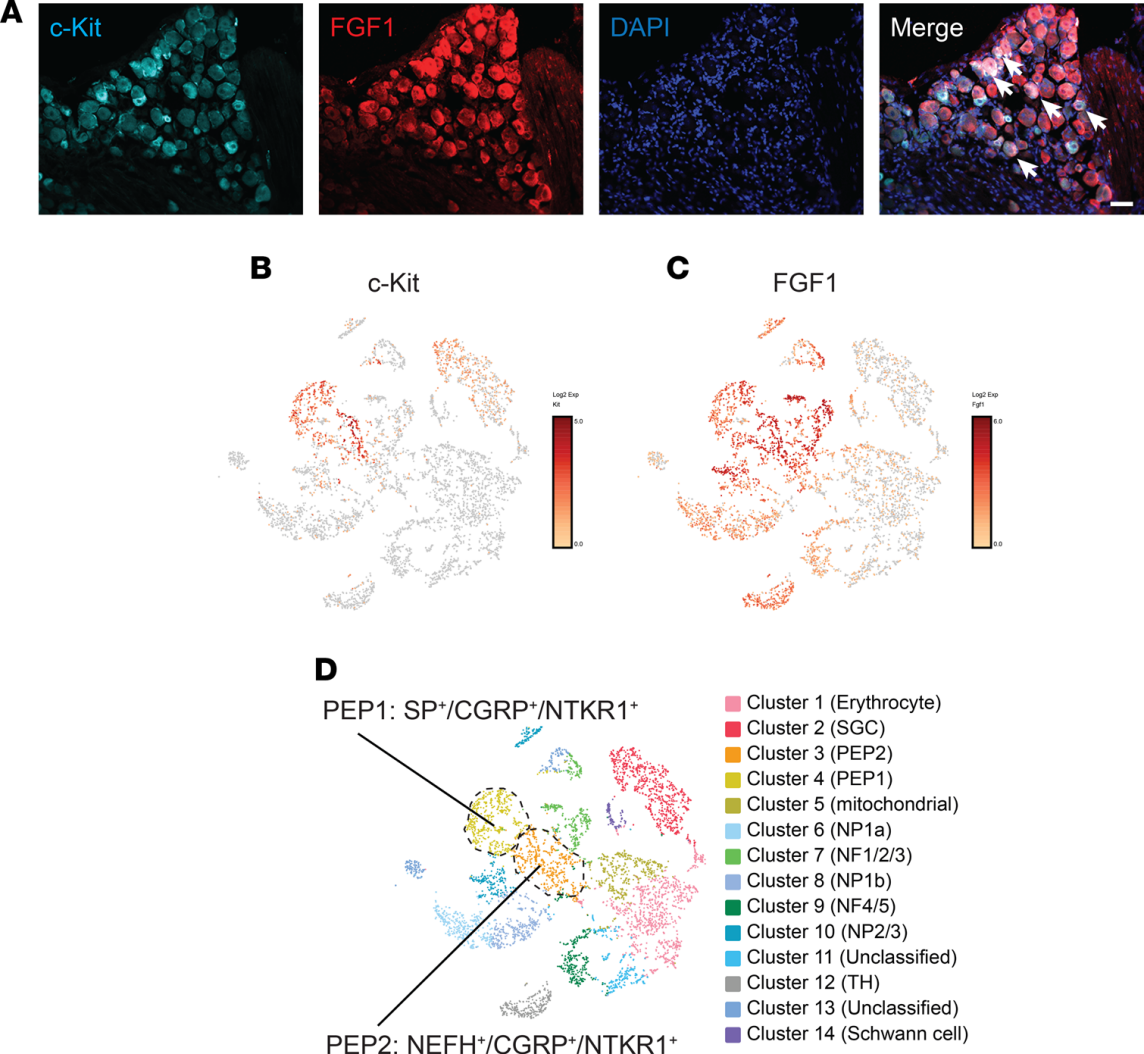
FGF1 is colocalized with c-Kit–expressing DRGs in mouse. (**A**) Representative IF image of colocalization among c-Kit, FGF1, and DAPI in murine DRGs. Original magnification, ×10; scale bar: 100 μm. Arrows indicate colocalization. (**B**–**D**) t-SNE plots of (**B**) c-Kit, (**C**) FGF1, and (**D**) cell cluster annotation in 10X Genomics Loupe Browser for single-cell RNA sequencing of L2–L5 DRGs of naive C57BL/6 mice (GSE325147).

**Figure 11:**
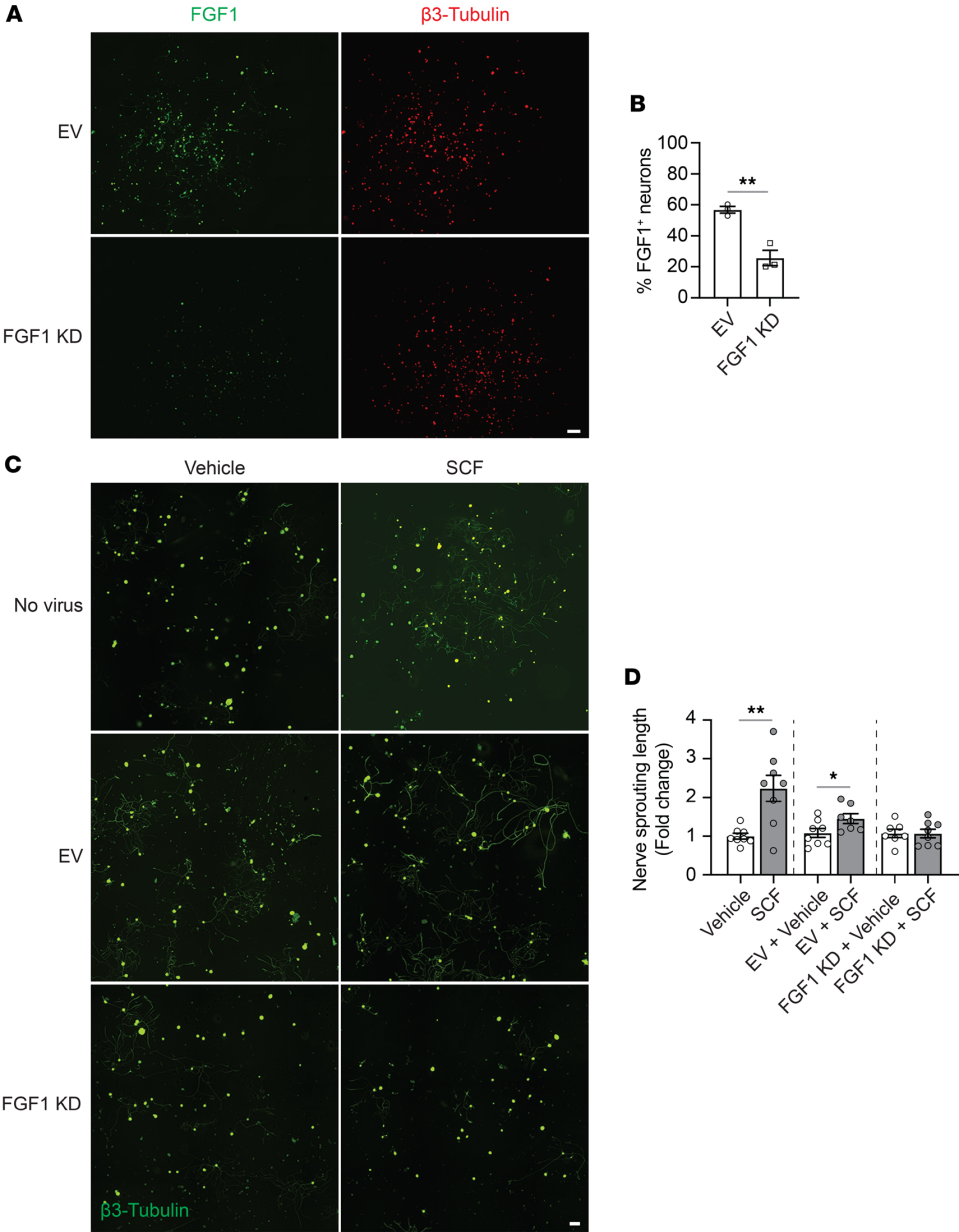
FGF1 is responsible for SCF-mediated nerve sprouting in vitro. (**A**) Representative IF images of FGF1 expression in murine primary DRG cells transfected with lentivirus containing empty vector (EV) or FGF1 knockdown (KD) shRNA. β3-Tubulin (a pan neuronal marker) was used as a positive control. Original magnification, ×10; scale bar: 100 μm. (**B**) Quantification of **A**. Mean ± SEM. Student’s *t* test (**P ≤ 0.01). (**C**) Representative IF images of β3-tubulin–positive nerve sprouting of murine primary DRG cells transfected with lentivirus containing EV or FGF1 KD shRNA with or without SCF. Original magnification, ×10; scale bar: 100 μm. (**D**) Quantification of **C**. Mean ± SEM. Student’s *t* test (*P ≤ 0.05, **P ≤ 0.01).

In an effort to elucidate the downstream mechanisms behind SCF-mediated nerve sprouting and CIBP, we treated murine DRGs with rmSCF ex vivo. While SCF treatment activated c-Kit, pathways known to be downstream of this axis (Akt, Erk, p38) were not activated (Figure 9A). To understand the downstream targets of the SCF/c-Kit axis, we performed proteomic analysis (PXD076785) and found that SCF treatment in the DRGs significantly enhanced 9 proteins (Psph, Clec21, Fgf1, Trafd1, Otog, Gsdme, Hes1, Smg1, Cnot4) (Figure 9B) and activated 7 proteins (Plekha4, Kmt2e, Atf7, Fgf1, Cplx1, Nsf, Myl7) (Figure 9C). Notably, only downstream FGF1, which is known for its role in the induction of nerve sprouting (40), was both enhanced and activated. We then further analyzed our proteomics data using Ingenuity Pathway Analysis, which revealed that SCF treatment activated pathways involved in nerve growth (Figure 9, D and E). We then performed Western blot analysis to validate the enhancement (Figure 9F) and activation (Figure 9G) of FGF1 by ex vivo rmSCF treatment in DRG tissues. FGF1 was colocalized with c-Kit–positive DRG neurons in 45.4% ± 2.6% of the FGF1- or c-Kit–positive cells (Figure 10A). Consistently, single-cell RNA sequencing (scRNA-seq) of DRG sensory neurons (GSE325147) revealed that the c-Kit–positive population (peptidergic clusters as shown in Figure 2), based on transcriptomic markers from prior studies (47), also expressed FGF1, although FGF1 expression is not c-Kit specific (Figure 10, B–D). Furthermore, when FGF1 was downregulated in primary DRG sensory neurons (Figure 11, A and B), SCF-mediated nerve sprouting was prevented ex vivo (Figure 11, C and D). These results suggest that FGF1 is responsible for the SCF/c-Kit axis-mediated nerve sprouting.

## Discussion

Herein, when cancer cells, known to induce CIBP, were inoculated intrafemorally into mice, c-Kit was activated in their DRGs. Its ligand, SCF, was highly expressed by cancer cells in both murine and patient bone metastatic tissues. Furthermore, in vitro cancer-derived SCF induced nerve sprouting, which is a known mechanism of CIBP (23–26), while c-Kit inhibitor treatment precluded this effect. Consistently, cancer-derived SCF induced nerve sprouting in vivo resulting in CIBP; however, cancer-derived SCF did not alter bone remolding or tumor growth in the bone. Moreover, SCF enhanced and activated FGF1 in murine DRGs. When FGF1 was knocked down in murine DRGs, SCF no longer induced nerve sprouting. Taken together, our data suggest that the SCF/c-Kit axis is responsible for induction of CIBP by enhancing sensory nerve sprouting through FGF1. Although SCF did not increase calcium influx in our study, it is established that abnormal nerve growth can contribute to the development of CIBP (23–26). While additional stimuli from other factors are necessary to induce pain sensation, we propose that SCF-mediated abnormal nerve growth increases nerve exposure to these stimuli, thereby facilitating the induction of CIBP. However, further research is warranted to clarify these mechanisms.

Multitargeted RTK inhibitors, which block c-Kit activation among other RTKs, have been tested clinically to treat patients with bone metastatic (NCT00410813; NCT00137436; and NCT00439270) (52–54), as the SCF/c-Kit axis is known to play an important role in the development of bone metastasis (55–58). Despite this, studies NCT00410813 and NCT00137436 have only had limited success in controlling tumor progression in bone, and these studies failed to include CIBP as a primary or secondary outcome (52, 53). However, patients with metastatic castration–resistant prostate cancer were found to have favorable response to combination therapy with docetaxel and dasatinib (NCT00439270) (54). In this phase I/II clinical study, 85% of the 46 patients had bone metastases and, among the evaluable responders, 92% had improved bone scans or no new lesion/CIBP at 18 weeks. Furthermore, 60% of the phase II patients had decreased CIBP using the Brief Pain Inventory from baseline measurement to cycle 6 (54). While clinical trials have shown some promise using c-Kit inhibition as treatment for bone metastasis, use of these inhibitors as interventions for CIBP remains understudied. Our study indicates that SCF levels do not influence tumor size or bone remodeling but rather affect CIBP behaviors. Therefore, blockade of the SCF/c-Kit axis using RTK inhibitors targeting c-Kit may offer therapeutic benefits for CIBP management without altering disease progression. Additional clinical research is clearly warranted to further validate these findings.

While clinical trials have not focused on c-Kit inhibition as a treatment for CIBP, preclinical studies have demonstrated the applicability of c-Kit inhibition as a therapy for CIBP in patients. In an animal study, treatment with a multitargeted RTK inhibitor, which blocks c-Kit activation among other RTKs, was found to significantly reduce CIBP (59). Additionally, our in vitro study using RTK inhibitors, which prevented LL/2 cell CM-induced nerve sprouting, further suggests the importance of c-Kit inhibition as a therapeutic avenue for CIBP. Unfortunately, as stated above, clinical trials using c-Kit inhibitors to treat bone metastasis have not adequately examined CIBP as an outcome, nor have they considered the contribution of tumor-derived SCF. Our in vivo data demonstrated that CIBP was enhanced in an SCF-dependent manner, suggesting that tumor SCF expression may need to be considered for treatment of CIBP. The next step of our investigation will, therefore, focus on assessing the effects of c-Kit inhibition in murine models intrafemorally implanted with cancer cells expressing elevated levels of SCF. Together, our findings and those of other studies (29, 39, 54, 59) suggest that c-Kit can be a potential target to treat CIBP and patients with high SCF expression in their tumors, specifically, could greatly benefit from c-Kit inhibition.

Selective inhibition of c-Kit expressed by sensory nerves could be challenging because the currently available c-Kit inhibitors are nonspecific and also target other RTKs (60). Moreover, several normal stem cells are known to express c-Kit, so blocking this receptor could impair hematopoiesis (61). Furthermore, c-Kit inhibition has been known to induce cytopenia, hypogonadism, painful gynecomastia, and hand-foot skin reaction in addition to other adverse events (62, 63). Thus, it is difficult to block the SCF/c-Kit axis by these c-Kit inhibitors without inducing severe side effects (64). Furthermore, these c-Kit inhibitors may not be effective for long-term therapy because they are also known to induce treatment resistance (61). Since, when FGF1 is knocked down, SCF no longer enhances nerve sprouting, FGF1 may serve as a novel therapeutic target in the quest to treat CIBP. Indeed, FGF1 has been previously implicated in the activation of neurons (65) as well as in the induction of neurite outgrowth (40, 66), and further studies are justified to further elucidate its role in CIBP.

In addition to FGF1’s role in nerve sprouting, another prospect worth considering is the role of FGF1 in oxidative damage and inflammation. It was previously found in a murine model of osteoarthritis, another bone-related condition that presents clinically with pain, that FGF1-KD reversed oxidative damage and inflammation (67). Both oxidative damage and inflammation are known for their roles in CIBP development (68–70) through the contribution of reactive oxygen species to inflammatory signaling pathways (71, 72). Moreover, FGF1 is predominately expressed in the central nervous system (brain stem, spinal cord, etc.) (73), whereas c-Kit is expressed more globally (hematopoietic stem cells, prostate, liver, etc.) (74). Therefore, although it is not feasible to entirely eliminate adverse effects from any therapy, FGF1 may be a safer therapeutic target due to decreased potential of off-target effects. Currently, many FGF receptor inhibitors and growth factor inhibitors have been tested clinically for treatment of cancer as FGFs, and their receptors are widely implicated in malignancies (75–77). Interestingly, patients with metastatic prostate cancer demonstrated significant improvement in proportion and duration of pain response following treatment with growth factor inhibitor suramin, a known FGF1 inhibitor (78). Collectively, this warrants further investigation into the efficacy and safety of FGF1 blockade in treatment for CIBP.

Treating CIBP presents as a major clinical challenge. Nearly half of patients with bone metastasis do not achieve adequate pain management under current therapies (e.g., opioid and NSAIDs) (79). Consistently, treatment with the NSAID carprofen showed only modest relief in spontaneous pain and mechanical hypersensitivity, although this did not reach significance for guarding behavior in our model (Supplemental Figure 1). Furthermore, these treatments are known to induce severe side effects, leaving much room for improvement of pain outcomes and adverse events. Combination therapy may hold the key to improving CIBP treatments. Median duration of CIBP was significantly improved in patients with prostate cancer with bone metastasis treated with somatostatin analog/dexamethasone/zoledronate combination therapy compared with zoledronate monotherapy (80), while only minor, controllable side effects were observed. Furthermore, combination treatment of bisphosphonate and external beam radiotherapy was able to significantly reduce mean bone pain scores with minimal side effects. Importantly, this combination was able to reduce patient opioid use from 84% at baseline to 24% after 3 months (81). The success of combined therapies holds promise for improving safety and efficacy for patients with CIBP and thus may enable SCF/c-Kit/FGF1-targeted treatments to be used in combination to improve outcomes.

In conclusion, our study revealed that cancer cell SCF expression correlates with nerve sprouting and CIBP through its receptor c-Kit and that FGF1 may act as a downstream component of the SCF/c-Kit axis. The SCF/c-Kit/FGF1 pathway may hold the key to unlock a potential novel and safer therapeutic avenue for CIBP, for which few currently exist. Thus, our study could be a breakthrough for developing CIBP treatments as means to improve quality of life for patients.

## Methods

### Additional methods.

For all additional methods, please refer to Supplemental Methods.

### Sex as a biological variable.

Understanding the roles of the SCF/c-Kit axis in CIBP development is the primary outcome and focus of these studies. We used melanoma cells, prostate cancer cells, and lung cancer cells as a model of cancer with low levels of SCF, model of cancer with inter¬mediate levels of SCF, and model of cancer with high levels of SCF, respectively. We needed to assess the effects of cancer-derived SCF on CIBP development in the same conditions (male mice were used owing to the nature of prostate cancer). Therefore, we did not investigate sex as a biological variable in the present study; however, in future studies, we will explore this variable as it may contribute to SCF-induced CIBP.

### Cell culture.

DU145, RM-1, TRAMP-C1, LL/2, B16-F10, 4T1, HEK293, and PWR-1E cells were purchased from the ATCC. B6MYC-Cap0 cells were a gift from Leigh Ellis’ laboratory at Harvard University in Cambridge, Massachusetts, USA. RM-1, B16-F10, and LL/2 cells were transformed to stably express GFP and firefly luciferase by transduction with a lentivirus (Lenti-GF1-CMV-VSVG) (82). For details, see Supplemental Methods.

### Intrafemoral injection mouse model.

Luciferase-expressing cancer cells were inoculated intrafemorally into mice using our well-established approach to establish tumor within the marrow (83). Thereafter, tumor growth, CIBP behaviors, and bone remodeling were measured and evaluated by blinded observers. For details, see Supplemental Methods.

### GSEA of patients with prostate cancer.

GSEA (v4.3.2) (45, 84) was used to measure enrichment of the “HP_BONE_PAIN” in a prostate cancer patient cohort from TCGA (*n* = 554) (project ID: TCGA-PRAD). Briefly, the Gene Cluster Text file (.gct) was generated from the TCGA prostate cancer patient dataset. To generate the categorical class file (.cls) patients were stratified into “high SCF” (*n* = 277) and “low SCF” (*n* = 277) expressing groups. Number of permutations for GSEA was set to 1,000, and for the Gene MatriX file (.gmx), the “HP_BONE_PAIN” gene list was used (HP:0002653). For the generation of the chip platform (.chip), the TCGA gene list was used. The GSEA platform determined significance and generated heatmap data using Morpheus software developed by the Broad Institute.

### Proteomics and phosphoproteomics analysis.

Fresh DRG tissues were dissected from C57BL/6 mice as described in nerve sprouting assay methods and were incubated with NG medium for 30 minutes at 37°C, 5% CO_2_. Subsequently, DRGs were treated with either vehicle or 200 ng/mL SCF for 10 minutes or 1 hour. Following PBS wash, 200 μL RIPA Lysis Buffer (G-Biosciences, catalog no. 786490) supplemented with Protease Inhibitor Cocktail (APExBIO, catalog no. K1007) and Phosphatase Inhibitor Cocktail (APExBIO, catalog no. K1015) was added to each sample. DRG tissues were then homogenized by pestle on ice and placed on 4°C shaker for 1 hour followed by 20 minutes centrifugation at 16,000*g* to remove debris. DRG lysates (200 μL) were further reduced and alkylated by adding 10 μL of 200 mM Tris(2-carboxyethyl)phosphine (TCEP) and incubation at 55°C for 1 hour, followed by addition of 10 μL of 375 mM iodoacetamide and further incubation for 1 hour at room temperature. The protein pellet was isolated by overnight precipitation with 1 mL of chilled acetone at –20°C and centrifugation. The protein was reconstituted in 100 μL of 50 mM triethylammonium bicarbonate. Following measurement of protein concentration by BCA, 100 μg protein was digested with 2.5 μg of sequencing-grade modified trypsin overnight at 37°C followed by labeling of protein fractions using the TMTpro 16-plex label reagent kit (Thermo Fisher Scientific, catalog no. A44521) according to the manufacturer’s protocol. All treatment groups included 5 biological replicates, and the tandem mass tag for each sample was as follows: vehicle controls, 126, 127N, 127C, 128N, and 128C; 5-minute SCF treatments, 129N, 129C, 130N, 130C, and 131N; and 1-hour SCF treatments, 131C, 132N, 132C, 133N, and 133C. 120 μL from each sample was taken and pooled, while 5 μL was taken separately for quality control to assess labeling efficiency. The samples were acidified to pH 3 by trifluoroacetic acid and purified using a 1cc C18 Sep-Pak cartridge. The sample was split into two fractions for (a) phosphopeptide enrichment and (b) high-pH reversed-phase (Hp-RP) peptide fractionation for global proteomics. Phosphopeptide enrichment was performed using a Fe-NTA Phosphopeptide Enrichment Kit (Thermo Fisher Scientific, catalog no. A32992) according to the manufacturer’s protocol. Hp-RP peptide fractionation was performed using a High pH Reversed-Phase Peptide Fractionation Kit (Thermo Fisher Scientific, catalog no. 84868) according to the manufacturer’s protocol. Peptides were prepared in 5% acetonitrile with 1% formic acid for liquid chromatography–mass spectrometry (LC-MS/MS) analysis.

To perform LC-MS/MS analysis, samples were analyzed on an Orbitrap Eclipse Mass Spectrometer (Thermo Fisher Scientific) coupled with a Vanquish Neo nano-UHPLC system (Thermo Fisher Scientific) via the FAIMS (high-field asymmetric waveform ion mobility spectrometry) Pro interface. Peptides were separated on a DNV P (1500 bar, 75 μm × 500 mm) column for 120 minutes employing linear gradient elution consisted of water (A) and 80% acetonitrile (B) both of which contained 0.1% formic acid. Data were acquired using synchronous precursor selection–MS3 (SPS-MS3) based TMT method. MS2 scans were acquired for peptide identification using top-speed data-dependent scan where maximum number of MS2 spectra were collected from fragmentation of selected precursor ions per 3 seconds of cycle time between adjacent survey spectra (MS1). MS3 scan was sequentially performed for relative quantitation by multi-notch MS3-based TMT method where significant MS2 ions were selected by SPS with assistance of real-time database search, which were fragmented to generate reporter ion peaks. The MS2–MS3 scan was repeated with precursor ion subsets isolated by FAIMS, with compensation voltage set to –35 eV, –45 eV, and –55 eV sequentially. Dynamic exclusion option was enabled and duration of which was set to 120 seconds. To identify proteins, spectra were searched against the UniProt mouse protein FASTA database (17,082 annotated entries, October 2021) using the Sequest HT search engine with the Proteome Discoverer v2.5 (Thermo Fisher Scientific). Search parameters were as follows: FT-trap instrument; parent mass error tolerance, 10 ppm; fragment mass error tolerance, 0.6 Da (monoisotopic); enzyme, trypsin (full); no. maximum missed cleavages, 2; variable modifications, +15.995 Da (oxidation) on methionine, +304.207 Da (TMTpro) on lysine and N-term, +79.966 (phospho) on serine, threonine, and tyrosine; static modification, +57.021 Da (carbamidomethyl) on cysteine.

Ingenuity Pathway Analysis (QIAGEN) was performed to unveil the pathway behavior due to the differentially expressed and phosphorylated proteins using the data from phospho-proteomics analysis. For the analysis, the differential expression was defined, and phosphorylation was set at a cutoff of *P* < 0.05 compared with vehicle. The species and sample type were set as “Mouse” and “Tissues and Primary cells,” respectively. Based on the results of core analysis, the significantly enriched pathways (*P* < 0.01) that are associated with neural physiological activities were ranked by *z* score.

### scRNA-seq and analysis.

A single-cell suspension for scRNA-seq was performed according to the 10X Genomics guidelines. Briefly, ipsilateral lumbar DRGs (L2–L5) were collected from 2 sham-inoculated mice in a tube containing HBSS on ice. Samples were pooled in order to increase cell number for sequencing. The resulting DRGs were processed to single-cell suspensions. Cells with >80% viability were loaded into wells of a 10X Chromium single-cell capture chip targeting a cell recovery rate of 2,000–4,000 cells. Single-cell gel beads in emulsion were created on a Chromium Single-Cell Controller, and scRNA-seq libraries were prepared using the Chromium Single-Cell 3’ Library and Gel Bead kit according to the manufacturer’s protocol (10X Genomics). Sequencing libraries were loaded at 1.3 pM on an Illumina NextSeq500 with High Output 150 cycle kit for paired-end sequencing using the following read length: 26 bp Read1, 8 bp i7 Index, 0 bp i5 Index, and 98 bp Read2. The Cell Ranger Single-Cell Software Suite v7.2 were used to perform sample demultiplexing, alignment, filtering, and unique molecular identifier counting. The data for each respective subpopulation were aggregated for direct comparison of single-cell transcriptomes. Low-quality cells identified as having less than 200 expressed genes were discarded. Cells with low viability were also removed if their proportions of mitochondrial gene expression were larger than 40%. t-SNE, K-means, and UMAP clustering were employed to reduce data dimensionality and to cluster cells based on global expression. Cell clusters were annotated based on cell-type markers from prior studies (47).

### Statistics.

Numerical data are expressed as the mean ± SEM. Two observers were blinded to experimental conditions when performing quantification of all images. Statistical analysis was performed using GraphPad Prism and SAS 9.4 statistical program (SAS Inc.) with significance at *P* ≤ 0.05. Outcome measures were transformed to satisfy the conditional normality assumption as needed. A 2-tailed unpaired *t* test or 1-way or 2-way ANOVA with Tukey’s or Dunnett’s post hoc test was used to compare single measurements between groups. For outcome measures (e.g., log-transformed radiance and adjusted guarding time) collected repeatedly over time, mixed-effects models were used to compare mean differences between groups over time. Group, time, and group-by-time interaction were included in the model. Animals were treated as a random effect. Contrasts were calculated to compare mean differences between groups at each time point.

### Study approval.

All human studies and all animal studies followed the Declaration of Helsinki and the Wake Forest University School of Medicine Institutional Animal Care and Use Committee guidelines, respectively. All animals were obtained from The Jackson Laboratory, and all animal studies were approved by the Institutional Animal Care and Use Committee at Wake Forest University School of Medicine (protocol A24-021 and A22-165). All human studies performed were approved by the Institutional Review Board at The Jikei University School of Medicine [IRB # 28-140(8383), 30-136(9157)]. Informed consent was obtained from all individuals involved in the current study.

### Data availability.

All data that support this study’s findings are available in the main text or supplemental materials. The corresponding values for all data points in graphs are reported in the Supporting Data Values file. Single-cell sequencing results have been deposited into Gene Expression Omnibus (GSE325147). The mass spectrometry proteomics data have been deposited to the ProteomeXchange Consortium via the PRIDE (85) partner repository with the dataset identifier PXD076785 and 10.6019/PXD076785. For other original data or detailed protocols, contact the corresponding author.

## Author contributions

KFC: conceptualization, methodology, validation, investigation, formal analysis, writing of the original draft, review and editing of the manuscript, and visualization. JO: conceptualization, methodology, validation, investigation, formal analysis, writing of the original draft, review and editing of the manuscript, and visualization. YY: methodology, validation, investigation, formal analysis, review and editing of the manuscript, and visualization. SHP: methodology, investigation, formal analysis, and review and editing of the manuscript. ST: investigation, resources, and review and editing of the manuscript. KR: investigation and review and editing of the manuscript. TMH: investigation and review and editing of the manuscript. JC: investigation and review and editing of the manuscript. LRS: investigation and review and editing of the manuscript. TK: resources and review and editing of the manuscript. JL: formal analysis, data curation, resources, and review and editing of the manuscript. CMF: formal analysis, data curation, resources, and review and editing of the manuscript. LDM: formal analysis, data curation, and review and editing of the manuscript. FCH: formal analysis and review and editing of the manuscript. YS: conceptualization, methodology, investigation, writing of the original draft, review and editing of the manuscript, visualization, supervision, project administration, and funding acquisition. KFC and JO contributed equally and share co–first authorship. The order of appearance was determined by their chronological involvement with the project.

## Conflict of interest

The authors have declared that no conflict of interest exists.

## Funding support

This work is the result of NIH funding, in whole or in part, and is subject to the NIH Public Access Policy. Through acceptance of this federal funding, the NIH has been given a right to make the work publicly available in PubMed Central.

- National Cancer Institute (R01CA238888 to YS; R21CA297068 to YS).
- Department of Defense (W81XWH-17-1-0541 to YS; W81XWH-19-1-0045 to YS).
- METAvivor (METAvivor Research Award to YS).
- Wake Forest Baptist Comprehensive Cancer Center Internal Pilot Funding (to YS).
- National Cancer Institute’s Cancer Center Support Grant award P30CA012197 to the Atrium Health Wake Forest Baptist Comprehensive Cancer Center.

## Supplementary Material

Supplemental data

Unedited blot and gel images

Supporting data values

## Version 1 05/05/2026

In-Press Preview

## Version 2 06/22/2026

Electronic publication
