# Supplementary material for: The contribution of stem cell factor and its receptor c-Kit to cancer-induced bone pain
Source: JCI Insight. 2026 May 5;11(12):e191905. doi: 10.1172/jci.insight.191905 (PMC13313531; doi:10.1172/jci.insight.191905)
Supplement: Supplemental data [file jciinsight-11-191905-s136.pdf]

Supplemental Materials

Title: The contribution of stem cell factor and its receptor c-Kit to cancer-induced bone pain through fibroblast growth factor 1

Authors: Kelly F. Contino[1*], Jenna Ollodart[1*], Yang Yu[1], Sun H. Park[1,2], Shunsuke Tsuzuki[1, 3], Kara Rollins[1], Tyler M. Heethouse[1], Joshua Chu[1], Laiton R. Steele[1,4], Takahiro Kimura[2], Jingyun Lee[5], Cristina M. Furdui[5], Lance D. Miller[1], Fang-Chi Hsu[6], and Yusuke Shiozawa[1]

[*]Co-first authors

Affiliations:

[1]Department of Cancer Biology and Atrium Health Wake Forest Baptist Comprehensive Cancer Center, Wake Forest University School of Medicine, Winston-Salem, NC, USA

[2]Department of Radiation Oncology, Wake Forest University School of Medicine, Winston-Salem, NC, USA

[3]Department of Urology, Jikei University School of Medicine, Tokyo, Japan

[4]Department of PA Studies, Wake Forest University School of Medicine, Winston-Salem, NC, USA

[5]Department of Internal Medicine, Section on Molecular Medicine, Wake Forest University School of Medicine, Winston-Salem, NC, USA

[6]Department of Biostatistics and Data Science, Wake Forest University School of Medicine, Winston-Salem, NC, USA

Corresponding authors:

Yusuke Shiozawa, MD, PhD

Wake Forest School of Medicine

Medical Center Blvd,

Winston-Salem, NC 27157

Phone: 336-716-8743; Fax: 336-716-0255; E-mail: yshiozaw@wakehealth.edu

Conflict of interest:

No conflict of interest exists for the authors.

Legend for Supplemental Figures

Supplemental Figure 1. Carprofen provides some reduction in CIBP-related behaviors.

(**A**) Von Frey hypersensitivity quantification, (**B**) Guarding behavior quantification, and (**C**) Tumor growth measured by bioluminescence imaging of mice intrafemorally inoculated with RM-1. Mice were treated with NSAID carprofen at Day 7, 11, and 15. Von Frey hypersensitivity and guarding behavior were measured pre- and 3 hours post-NSAID carprofen treatment. Mean ± SEM. Student's t-test ($^{*}p \leq 0.05$, $^{**}p \leq 0.01$).

Supplemental Figure 2. Additional representative images demonstrating SCF enrichment in bone metastatic cancer cells in the marrow.

Representative IF images of colocalization between cytokeratin-8 (CY8) and SCF in (**A**) bone marrow autopsy samples from prostate cancer patients who died from other causes (Patients w/o bone mets, n = 4) or bone metastases (Patients w/ bone mets, n = 5); (**B**) bone marrow of immunodeficient mice intrafemorally inoculated with HBSS (Sham mice) and DU145 (DU145-bearing mice) at 54 days post-tumor inoculation; and (**C**) bone marrow of immunocompetent mice intrafemorally inoculated with HBSS (Sham mice) and RM-1 (RM-1-bearing mice) at 21 days post-tumor inoculation. Magnification 10x. Bar = 100µm for IF images. DAPI is used for nuclear staining.

Supplemental Figure 3. Validation of SCF and cytokeratin-8 antibody specificity.

Representative IF in vitro images of colocalization between cytokeratin-8 (CY8) and SCF in (**A**) LL/2 EV and SCF KD and (**B**) LL/2 EV and primary bone marrow cultured cells. Magnification 40x. Bar = 50µm. DAPI is used for nuclear staining.

Supplemental Methods

Study Approval

All human studies and all animal studies followed the Declaration of Helsinki and the Institutional Animal Care and Use Committee Guidelines, respectively. All animal studies were approved by the Institutional Animal Care and Use Committee at Wake Forest University School of Medicine (Protocol A24-021 and A22-165). All human studies performed were approved by the Institutional Review Board at Jikei University School of Medicine [IRB # 28-140(8383), 30-136(9157)]. Informed consent was obtained from all subjects involved in the current study.

Sex as a Biological Variable

Understanding the roles of the SCF/c-Kit axis in CIBP development is the primary outcome and focus of these studies. We used melanoma cells, prostate cancer cells and lung cancer cells as a model of SCF low cancer, SCF intermediate cancer and SCF high cancer, respectively. We needed to assess the impacts of cancer-derived SCF on CIBP development in the same conditions (male mice due to the nature of prostate cancer). Therefore, we did not investigate sex as a biological variable in the present study; however, in future studies, we will explore this variable as it may contribute to SCF-induced CIBP.

Cell Culture

Murine prostate cancer cell lines RM-1 (American Type Culture Collection (ATCC), Cat. #: CRL-3310), TRAMP-C1 (ATCC, Cat. #: CRL-2730); murine Lewis lung carcinoma cell line LL/2 (ATCC, Cat. #: CRL-1642); murine melanoma cell line B16-F10 (ATCC, Cat. #: CRL-6475); murine breast cancer cell line 4T1 (ATCC, Cat. #: CRL-2539); human prostate cancer cell line

DU145 (ATCC, Cat. #: HTB-81); human prostate epithelial cell line PWR-1E (ATCC, Cat. #: CRL-3661); and human embryonic kidney cell line HEK293 (ATCC, Cat. #: CRL-1537) were purchased from ATCC. B6MYC-Cap0 were a generous gift from Dr. Leigh Ellis' laboratory at Harvard University. RM-1, LL/2, B16-F10, B6MYC-Cap0, DU145, and HEK293 were maintained in Dulbecco's Modified Eagle's medium (DMEM, Thermo Fisher Scientific (Gibco), Cat. #: 11-995-073). 4T1 cells were maintained in Roswell Park Memorial Institute (RPMI) 1640 medium (Thermo Fisher Scientific (Gibco), Cat. #: 11-875-093). TRAMP-C1 were maintained in DMEM adjusted to contain 4mM L-Glutamine (Thermo Fisher Scientific (Gibco), Cat. #: 25-030-164), 1.5g/L sodium bicarbonate (Millipore Sigma, Cat. #: S5761-500G) 4.5g/L glucose (Millipore Sigma, Cat. #: 11995065), supplemented with 0.005mg bovine insulin (Millipore Sigma, Cat. #: I6634-50MG) and 10nM dehydroepiandrosterone (Millipore Sigma, Cat. #: D-063-1ML). All cultures were supplemented with 10% (V/V) fetal bovine serum (FBS, Thermo Fisher Scientific (Gibco), Cat. #: 26-140-079), 1% (V/V) penicillin-streptomycin (Thermo Fisher Scientific (Gibco), Cat. #: 15-140-163), and 1% (V/V) L-Glutamine (Thermo Fisher Scientific (Gibco), Cat. #: 25-030-164). Cells were incubated at 37°C, 5% CO_2, and 100% humidity, and were routinely passaged when no more than 80% confluent.

Prior to the animal studies, some cancer cell lines (RM-1, B16-F10, LL/2) were transformed to stably express green fluorescent protein (GFP) and firefly luciferase by transduction with a lentivirus (Lenti-GF1-CMV-VSVG) generated by the University of Michigan Vector Core [1]. The transduced cells were sorted for GFP positive cells at the Wake Forest Baptist Comprehensive Cancer Center Flow Cytometry Shared Resource using an Astrios EQ (Beckman Coulter), expanded and frozen at low passage (<10).

Intrafemoral Injection Mouse Model

Luciferase-expressing cancer cells were intrafemorally inoculated into mice using our well-established approach to establish tumor within the marrow [2]. Briefly, an incision was made in the right hind limb skin to expose the muscle. Then, the rectus femoris and vastus medialis muscles were separated, and the rectus femoris and patella were moved to expose the femur condyles. A 27G needle was used to create the hole in the femur and then replaced with C313I injector (Plastics One, Inc., Cat. #: C313I). After confirming that the injector was in the intramedullary space by an X-ray (Faxitron Bioptics MultiFocus X-ray system), cancer cells [suspended in 5-10μL of Hanks buffered saline solution (HBSS) (Thermo Fisher Scientific (Gibco), Cat. #: 14175103)] were injected using a 10μL Hamilton syringe (Hamilton Company, Cat. #: 80300) attached to tygon tubing. The injection site was plugged with bone cement to delay spread of the tumor into the adjacent soft tissue. The patella was then gently returned to its correct orientation, and muscles were secured back into position using a horizontal mattress suture technique and 7-0 absorbable sutures (Patterson Veterinary, Cat. #: 07-809-2011). Wound closure was then achieved with the same sutures. The same surgical procedure was used for sham animals except that same amount of HBSS was injected instead of cancer cells. Thereafter, tumor growth, CIBP behaviors, and bone remodeling were measured.

Measure of Tumor Growth

IVIS Lumina LT Series III (PerkinElmer) was used to obtain bioluminescent images (BLI) through the Cell and Viral Vector Core Laboratory of the Wake Forest Baptist Comprehensive Cancer Center. Briefly, 30mg/ml luciferin (PerkinElmer, Cat. #: 122799) was delivered by intraperitoneal injections to luciferase-expressing cancer cell-bearing mice, and ventral images were taken 12 min

post-injection under 2.5% isoflurane/air anesthesia. The total tumor burden of each animal was calculated using regions of interest (ROI) that encompassed the entire tumor area in the femur.

Measure of Cancer-Induced Bone Pain Behavior

Twice weekly, guarding behavior measurements were performed as previously described [3, 4]. Tumor-bearing mice demonstrate a progressive increase in spontaneous pain behaviors, including guarding and periodic flinching of the inoculated limb which is not observed in sham inoculated mice. Briefly, mice were placed on nylon mesh platforms in clear plastic enclosures in a de-identified manner and acclimated for 30 min. Mice were recorded for 5 min, and video recordings were provided to blinded experimenters for guarding time analysis. Mice were also subjected to Von Frey assay up-down method to measure mechanical hypersensitivity by a blinded experimenter as previously described [5, 6]. Briefly, mice were placed on nylon mesh platforms in clear plastic enclosures and acclimated for 30 minutes. Filaments of increasing stiffness were applied to the plantar aspects of the hindpaw until fibers bend slightly for a maximum of 6 seconds. Personnel were blinded to treatment groups. Withdrawal or licking of the hindpaw after application will be recorded as a positive response. In order to test reversal of cancer-induced bone pain behaviors, we injected mice subcutaneously into the intrascapular space with NSAID carprofen (Thermo Fisher Scientific, Cat. #: C27015G) at a dose of 25mg/kg as previously described [7]. After 2.5 hours, mice were then placed on the nylon mesh platforms in clear plastic enclosures and acclimated for 30 min. Guarding behavior and Von Frey were then performed, respectively to measure cancer-induced bone pain post-carprofen treatment.

Measure of Bone Remodeling

Digital cabinet Faxitron Bioptics MultiFocus X-ray system was employed to x-ray mice at least once each week. The X-ray images were then provided to a blinded observer to perform longitudinal scoring of study endpoints using the following scales for assessing the extent of bone destruction [8]: 0 = Bones with no lesions; 1 = Bones with one to three small pits of radiolucent lesions; 2 = Bones with three to six small pits of radiolucent lesions; 3 = Bones with obvious loss of medullary bone and erosion of cortical bone; 4 = Bones with full thickness unicortical bone loss; and 5 = Bones with full thickness bicortical bone loss and displaced skeletal fracture.

Phospho-Specific Protein Microarray Analysis

To determine which proteins were tyrosine-phosphorylated differentially in the sensory nerve of RM-1-inoculated mice (n = 11) and sham-inoculated mice (n = 12), lumbar DRGs (L2-L5) were collected and subjected to protein extraction. These samples were then labeled with biotin and hybridized on Tyrosine Phosphorylation ProArray (Full Moon Biosystems, Cat. #: PST228). This array can detect 228 tyrosine phosphorylation sites and contains 6 technical replicates for each phosphorylation site. The conjugated labeled protein was detected using Cy3-streptavidin. Reading of slides and data analysis of the Phosphorylation ProArray was performed by Full Moon Biosystems.

IHC and IF of Mouse Bone Tissues

At the termination of animal experiments, mice were perfused with 4% PFA. Femurs were dissected and fixed in 10% formalin (Thermo Fisher Scientific, Cat. #: SF100-4) for 24 hrs at 4°C. Tissue was then decalcified with 10% Ethylenediaminetetraacetic acid (EDTA) (Thermo Fisher

Scientific (Thermo Scientific Chemicals), Cat. #: AC327200025), in pH 7.4 PBS for 14 days (the decalcification solution was replaced on day 7) at 4°C. Thereafter, femurs were embedded in paraffin or optimal cutting temperature (O.C.T.) compound (Thermo Fisher Scientific, Cat. #: 23-730-571) and then cut into 8-μm-thick sections or 10-μm-thick sections, respectively. For IHC staining, slides were blocked in 0.3% endogenous peroxidase for 10 min at room temperature, washed 2 times in 0.3% 1x DPBS, blocked in rabbit serum for 1 hr, and incubated with anti-SCF primary antibody (Abcam, Cat. #: ab64677) overnight at 4°C. Slides were then incubated with biotinylated anti-rabbit IgG secondary antibody SS Rabbit Link (BioGenex, Cat. #: HK336-5R) for 1 hr at room temperature. Antibody detection was performed using VECTASTAIN ABC kit (Vector Laboratories, Cat. #: PK-6100). The SCF positive area (DAB intensity) was visualized using a Nikon Eclipse Ni fluorescent microscope. For IF staining, mouse bone samples were blocked with 3% normal donkey serum (Jackson Immunoresearch, Cat. #:017-000-121) and 0.3% Triton X-100 (Millipore Sigma, Cat. #: X100-500ML) in 0.01M PBS for 1 hr at room temperature. Then, the slides were incubated with anti-cytokeratin8 primary antibody (1:200, Abcam, Cat. #: 84467) and anti-SCF primary antibody (1:200, Santa Cruz, Cat. #: sc-13126) or anti-PGP9.5 (Cedarlane Labs, Cat. #: CL7756AP-50) overnight at 4°C. The cells were then labeled with the secondary antibodies [CY3-conjugated donkey anti-mouse IgG antibody (1:500, Jackson Immunoresearch, Cat. #: 715-165-150), CY5-conjugated donkey anti-rabbit IgG antibody (1:400, Jackson Immunoresearch, Cat. #: 711-175-152)] for 2 hrs at room temperature. After washing 4 times with 1x DPBS, the cells were mounted with ProLong^{TM} Gold antifade mountant with DAPI and imaged using FV4000 laser scanning confocal microscope. IF images were pseudo-colored to accommodate color-blindness. All analysis was performed using ImageJ.

IHC and IF of Human Bone Autopsy Samples

Autopsy bone samples obtained from prostate cancer patients with bone metastases and patients without bone metastasis were collected between 2011-2015 and sectioned at Jikei University School of Medicine (5µm thickness) [patients without bone metastasis (n = 3) and prostate cancer patients with bone metastasis (n = 2)] Before incubating with the primary antibody, slides from patient samples were baked for 90 min at 60°C and treated with DeCal Retrival solution (Biogenex, HK089-5K) for antigen retrieval for 30 min at room temperature. Slides were then subject to either IHC or IF. For IHC staining, slides were blocked in 0.3% endogenous peroxidase for 10 min at room temperature, washed 2 times in 0.3% 1x DPBS, blocked in rabbit serum for 1 hr, and incubated with anti-SCF primary antibody (Abcam, Cat. #: ab64677) overnight at 4°C. Slides were then incubated with biotinylated anti-rabbit IgG secondary antibody SS Rabbit Link (BioGenex, Cat. #: HK336-5R) for 1 hr at room temperature. Antibody detection was performed using VECTASTAIN ABC kit (Vector Laboratories, Cat. #: PK-6100). The SCF positive area (DAB intensity) was visualized using a Nikon Eclipse Ni fluorescent microscope. For IF staining, patient samples were blocked with 3% normal donkey serum (Jackson Immunoresearch, Cat. #: 017-000-121) and 0.3% Triton X-100 (Millipore Sigma, Cat. #: X100-500ML) in 0.01M PBS for 1 hr at room temperature. Then, the slides were incubated with anti-cytokeratin8 primary antibody (1:200, Abcam, Cat. #: 84467) and anti-SCF primary antibody (1:200, Santa Cruz, Cat. #: sc-13126) overnight at 4°C. The cells were then labeled with the secondary antibodies [CY3-conjugated donkey anti-mouse IgG antibody (1:500, Jackson Immunoresearch, Cat. #: 715-165-150), CY5-conjugated donkey anti-rabbit IgY antibody (1:400, Jackson Immunoresearch, Cat. #: 711-175-152)] for 2 hrs at room temperature. After washing 4 times with 1x DPBS, the slides were mounted with ProLong™ Gold antifade mountant with DAPI and imaged using FV4000 laser scanning

confocal microscope. IF images were pseudo-colored to accommodate color-blindness. All analysis was performed using ImageJ.

IF of Murine DRG Tissue

At the termination of animal experiments, mice were perfused with 4% PFA. Lumbar DRGs (L2-L5) were dissected and fixed in 10% formalin for 24 hrs at 4°C. Thereafter, DRGs were embedded in O.C.T. compound, and then cut into 16-µm-thick sections. IF for c-Kit, substance P, CGRP, and IB4 was performed on fixed DRG tissue. After blocking with 5% normal donkey serum in 0.03% Triton X-100 in PBS for 1 hr at room temperature, the cells were incubated with primary antibodies [anti-c-Kit antibody (1:400, R&D Systems, Cat. #: AF1356), anti-substance P antibody (1:400, ImmunoStar, Cat. #: 20064), anti-CGRP antibody (1:3000, Millipore Sigma, Cat. #: C8198-25UL), and/or anti-IB4 antibody (1:1000, Millipore Sigma, Cat. #: L2140-.2MG)]. The cells were then labeled with the secondary antibodies [CY3-conjugated donkey anti-rabbit IgG antibody (1:500, Jackson Immunoresearch, Cat. #: 711-165-152), CY5-conjugated donkey anti-chicken IgG antibody (1:400, Jackson Immunoresearch, Cat. #: 703-175-155), or CY2-conjugated donkey anti-mouse IgG antibody (1:600, Jackson Immunoresearch, Cat. #: 715-225-150)] for 2 hrs at room temperature. After washing 5 times with 1x DPBS, the cells were mounted with ProLong™ Gold antifade mountant with DAPI. All images were taken using a Nikon Eclipse Ni fluorescent microscope system.

Nerve Sprouting Assay

Nerve sprouting assays were performed as previously described by our group [9]. Concisely, a single-cell suspension of murine primary DRG sensory neurons were obtained from the lumbar

DRGs (L2-L5) of male C57BL/6 mice (8-12 weeks of age) through multiple enzymatic digestions and density gradient centrifugation. Then, 500-1,000 DRG cells in 30µL of warm neuronal growth (NG) medium [Neurobasal-A (Thermo Fisher Scientific (Gibco), Cat. #: 21-103-049), 1% N2 (Thermo Fisher Scientific (Gibco), Cat. #: 17502048), 2% B-27 (Thermo Fisher Scientific (Gibco), Cat. #: 17-504-044), 2mM L-glutamine, 1% penicillin-streptomycin, and 0.4% glucose] were seeded onto the center of 14mm round coverslips (MatTek Corp., Cat. #: P12G-1.5-14-F), pre-coated with 50µg/mL Poly-D-lysine overnight at 4°C (Thermo Fisher Scientific (Gibco), Cat. #: A3890401) and 20µg/mL laminin for 1 hr at 37°C (Thermo Fisher Scientific (Corning), Cat. #: CB-40232), in 24-well plate. After 1-2 hrs, 1mL of warm NG medium was used to gently flood each well, then cells were maintained at 37°C with 5% CO_2. After 48 hrs of DRG neuronal culture establishment, half (500µL) of NG medium was replaced with 500µL of either control or cancer cell-derived CM, and then the DRG cells were incubated for another 48-72 hrs. In some case, the DRG cells were treated with vehicle or recombinant mouse SCF (50ng/mL, Miltenyi Biotec, Cat. #: 130-101-693). In other experiments, DRG cells were treated with cancer-cell derived CM and either vehicle or c-Kit inhibitor ISCK03 (10nM, Selleck Chemicals, Cat. #: S2070) or Ripretinib (5µM, Selleck Chemicals, Cat. #: S8757). At the termination of experiments, DRG cells were fixed in 500µL of 4% PFA (Thermo Fisher Scientific, Cat. #: AA433689M) overnight at 4°C and subjected to immunofluorescence staining or stored in 1x DPBS at 4°C until use. For each coverslip, a large-scan image of the coverslip was taken using the Nikon Eclipse Ni fluorescent microscope system (Nikon). Images were analyzed using Visiopharm or ImageJ (NIH) software, respectively.

IF of Murine Primary DRG Cells

IF for β3-tubulin (TUBB3), c-Kit, FGF1, substance P, CGRP, and IB4 was performed on fixed primary DRG cells. After blocking with 5% normal donkey serum in 0.03% Triton X-100 in PBS for 1 hr at room temperature, the cells were incubated with primary antibodies [anti-TUBB3 antibody (1:1000, Biolegend, Cat. #: 801201), anti-PGP9.5 antibody (Cedarlane Labs, Cat. #: CL7756AP-50), anti-c-Kit antibody (1:400, R&D Systems, Cat. #: AF1356), anti-FGF1 antibody (1:400, Thermo Fisher Scientific, Cat. #: PA5-115225), anti-substance P antibody (1:400, ImmunoStar, Cat. #: 20064), anti-CGRP antibody (1:3000, Millipore Sigma, Cat. #: C8198-25UL), and/or anti-IB4 antibody (1:1000, Millipore Sigma, Cat. #: L2140-.2MG)] overnight at 4°C. The cells were then labeled with the secondary antibodies [CY3-conjugated donkey anti-rabbit IgG antibody (1:500, Jackson Immunoresearch, Cat. #: 711-165-152), CY5-conjugated donkey anti-chicken IgG antibody (1:400, Jackson Immunoresearch, Cat. #: 703-175-155), or CY2-conjugated donkey anti-mouse IgG antibody (1:600, Jackson Immunoresearch, Cat. #: 715-225-150)] for 2 hrs at room temperature. After washing 5 times with 1x DPBS, the cells were mounted with ProLong^{TM} Gold antifade mountant with DAPI. All images were taken using a Nikon Eclipse Ni fluorescent microscope system. Images were analyzed using Visiopharm or ImageJ (NIH) software, respectively.

Calcium Imaging

DRG cells were dissected and cultured as described above. DRG cells were pre-treated with either vehicle or 50ng/ml SCF for 24 hrs. To load the calcium indicator, cells were incubated with 1μM Fura-2 AM solution (Thermo Fisher Scientific, Cat. #: F1201) diluted in NG media at 37°C in the dark for 45 min. Following a wash of NG media, cells were further incubated with NG media at

37°C in the dark for 30 min. Before calcium imaging, the NG media was replaced with HBSS with Ca^{2+} and Mg^{2+}. The videos were taken by a FV4000 laser scanning confocal microscope (Olympus Corporation) with a 10× objective. The excitation wavelength was set as 408nm while the detected emission wavelength was set at 510nm. During recording, high potassium buffer (Millipore Sigma, Cat. #: P5655-100G) was added into HBSS to make a final potassium concentration of 50mM in order to depolarize the cells. The alteration of fluorescence intensity in DRG cells were video recorded and analyzed by ImageJ. These alterations of fluorescence intensity for each cell were normalized by each cell's own baseline fluorescence intensity. The normalized fluorescence intensity alteration over time was used for calcium imaging quantification.

Lentivirus Generation

5 x 10^6 HEK293T cells were seeded in 75mL flasks in 10mL DMEM supplemented with 10% FBS, 1% penicillin-streptomycin, and 1% L-Glutamine and maintained at 37°C with 5% CO_2. 24 hrs after passaging the cells, they were transfected with Lipofectamine 3000 (Thermo Fisher Scientific (Invitrogen), Cat. #: L3000008) and accompanying reagent p3000 according to manufacturer protocol. Expression plasmids used for transfections included EV (Millipore Sigma, Cat. #: SHC001), SCF shRNA (Millipore Sigma, Cat. #: NM_013598, Clone ID: TRCN0000305053), SCF OE (SinoBiological, Cat. #: MG50487-UT, RefSeq: NM_013598.1), and FGF1 shRNA (Millipore Sigma, Cat. #: NM_010197, Clone ID: TRCN0000066699), and packaging vectors included psPAX2 (9μg) (Millipore Sigma, Cat. #: SHP001) and pMD2.G (0.9μg) (Millipore Sigma, Cat. #: SHP001). Packaging plasmids in their respective concentrations were mixed with 9μg EV or SCF shRNA, SCF OE, FGF1 shRNA and 37.8μL P3000 and brought to a final volume of 500μL with opti-MEM reduced serum media (Thermo Fisher Scientific

(Gibco), Cat. #: 31985062). This mixture was then incubated for 5 min at room temperature. Lipofectamine 3000 (30µL) was mixed with 470µL opti-MEM and incubated at room temperature for 5 min. These two mixtures were then combined resulting in a final volume of 1mL. The mixture was then added to HEK293T cells following a 15 min incubation at room temperature. After 24 hr, cells were refed with 10mL of fresh DMEM supplemented with 10% FBS, 1% penicillin-streptomycin, and 1% L-Glutamine. The cell culture medium was then harvested after 48 hrs, filtered through a 0.45µm filter and stored at 4°C. The next day the virus was concentrated to 15x using an Amicon Ultra-15 centrifugal filter tube (Millipore Sigma, Cat. #: UFC905024), performed at 4000 x g for 20 min at room temperature. Virus was frozen at -80°C until use.

Lentiviral Transduction of Cancer Cells and DRGs

Luciferase-expressing LL/2, RM-1, and B16-F10 cells were seeded in 6-well plates at 2.5 x 10^5 cells per well in DMEM respectively with 10% FBS, 1% penicillin-streptomycin, and 1% L-Glutamine. Cells were transduced with EV, SCF KD, or SCF OE 1x lentivirus and 6µg/mL polybrene (Millipore Sigma, Cat. #: TR1003G) was added to enhance the transduction. One-two wells in each six-well plate were used as control wells. Cells were screened using 2µg/mL puromycin (Gemini Bio-Products, Cat. #: 400-128P-100). Once all cells in the control wells died, EV, SCF KD, and SCF OE cells were subjected to quantitative real time PCR (RT-qPCR) and enzyme-linked immunosorbent assay (ELISA) analysis to ensure knockdown or overexpression efficiency. For DRG cell transduction, EV or FGF1 1x lentivirus and 6µg/mL polybrene were added to DRG cell culture concurrently with NG media. Knockdown was validated using IF staining for FGF1 as previously described. All pipette tips, flasks, etc. that came in contact with virus were cleaned with bleach.

Real Time Quantitative PCR (RT-qPCR)

Cells or DRG tissues were lysed, and RNA was collected with RNeasy Mini Kit (Qiagen, Cat. #: 74104) according to manufacturer instructions. RNA concentrations were determined using the Nanodrop 1000 Spectrophotometer v3.8.1 (Thermo Fisher Scientific) and subsequently normalized between samples prior to first-strand cDNA synthesis. First-strand cDNA was synthesized using 0.5µg of total RNA using Invitrogen SuperScript II Reverse Transcriptase (Thermo Fisher Scientific (Invitrogen), Cat. #: 18064022). RT-qPCR was performed using TaqMan Gene Expression Master Mix (Applied Biosystems, Cat. #: 4369016) and the following primers: Mm99999915_g1 (*Gapdh*, mouse GAPDH); Mm00442972_m1 (*kitl*, mouse kit ligand (SCF); Mm00445212_m1 (*kit*, mouse c-Kit); Mm00490378_m1 (*Gas6*, mouse Gas6); Mm00437221_m1 (*Axl*, mouse Axl); Mm00439016_m1 (*flt3*, mouse FLT3); or Mm00442801_m1 (*flt3l*, mouse FLT3 ligand). The PCR reaction was run for 49 cycles (95°C for 15 sec and 60°C for 1 min) after an initial single cycle of 50°C for 2 min and 95°C for 10 min using CFX96 Touch™ Real-Time PCR Detection System (Bio-Rad). *Gapdh* was used as the reference gene.

Conditioned Medium Collection

To collect the conditioned medium (CM) from control or cancer cells, approximately 5 x 10^5 cells were seeded onto 10cm dishes in complete growth medium. After 24 hrs, the medium was replaced with 10mL of serum free corresponding growth medium. For the control CM, 10mL of serum free DMEM or RPMI medium were added to a 10cm dish without adding any cancer cells. After 24 hrs of incubation at 37°C with 5% CO_2, the CM was collected, filtered through a 0.2µm syringe filter (Millipore Sigma, Cat. #. CLS431222) to remove any cell debris and then stored at -20°C until use. Upon collection of CM, adherent cells were counted, and volume of media was

normalized.

Enzyme-linked Immunosorbent Assay (ELISA)

Bone marrow supernatant and cancer cell CM were collected with protease inhibitors and stored at -80°C. Briefly, bone marrow supernatant was collected by cutting off the distal and proximal epiphyses of mouse femurs following dissection. Next, bones were placed in 0.6mL microcentrifuge tubes (Thermo Fisher Scientific, Cat. #: 05-408-120) that had a hole cut into the bottom. The 0.6mL tube was then placed into a 1.5mL microcentrifuge tube (Thermo Fisher Scientific, Cat. #: 05-408-129) and secured with parafilm (Thermo Fisher Scientific, Cat. #: 13-374-10). Tubes were then centrifuged at 18,000 x g speed for 10 sec to separate bone marrow from femur. The concentration of SCF was measured using a commercially available SCF ELISA according to the manufacturer's protocol (R&D Systems, Cat. #: MCK00). For bone marrow supernatant samples, the concentration of FL was also measured using a commercially available FL ELISA according to the manufacturer's protocol (R&D Systems, Cat. #: MFK00).

Western Blotting

Fresh DRG tissues were dissociated from C57BL/6 mice as described in nerve sprouting assay methods and were subjected to the incubation with NG medium for 30 min at 37°C, 5% CO_2. Subsequently, DRG were treated with either vehicle, 200ng/mL SCF for 10 min, or 1 hr. Following PBS wash, 200µL RIPA Lysis Buffer (G-Biosciences, Cat. #:786490) supplemented with Protease Inhibitor Cocktail (APExBIO, Cat. #: K1007) and Phosphatase Inhibitor Cocktails (APExBIO, Cat. #: K1015) was added into each sample. DRG tissues were then homogenized by pestle on ice and placed on 4°C shaker for 1 hr followed by 20 min centrifugation at 16,000 x g to remove debris.

The supernatant of lysate was quantified by Pierce™ BCA Protein Assay Kits (Thermo Scientific, Cat. #: 23225) and diluted into the same concentration with lysis buffer containing 1x Laemmli SDS Sample Buffer (Thermo Scientific, Cat. #: AAJ61337AD) followed by 5 min boiling at 95°C. The denatured protein lyse were loaded into SDS-PAGE 10% Tris-Glycine Mini Protein Gels (Thermo Fisher Scientific (Invitrogen), Cat. #: XP00100BOX) and transferred to a 0.2μm PVDF membrane (Millipore Sigma, Cat. #: ISEQ00010). After blocking with 5% Bovine Serum Albumin (Thermo Scientific, Cat. #: BP9703100) in TBS Tween-20 Buffer (Thermo Fisher Scientific, Cat. #: 28360) for 1 hr, membrane was incubated with the following primary antibodies at 4°C overnight: anti-c-Kit (phospho Y703) antibody (1:500, Abcam, Cat. #: ab62154); anti-c-Kit (D13A2) antibody (1:500, Cell Signaling Technology, Cat. #: 3074); anti-FGF1 antibody [EPR19989] (1:1000, Abcam, Cat. #: ab207321); or anti-GAPDH antibody (1:1000, Cell Signaling Technology, Cat. #: 2118), Thereafter, blots were incubated with anti-rabbit IgG HRP-conjugated secondary antibody (1:5000, Cell Signaling Technology, Cat. #: 7074S) at room temperature for 1 hr. Protein expression was detected with Pierce™ ECL Western Blotting Substrate (Thermo Fisher Scientific, Cat. #: 32209). To detect the phosphorylation of FGF1, 500μg/sample protein lysate were subjected to phosphorylation enrichment using Pierce Phosphoprotein Enrichment Kit (Thermo Fisher Scientific, Cat. #: 90003). Enriched phosphorylated protein samples were subjected to FGF1 detection using western blotting as described above.

Gene Set Enrichment Analysis of Prostate Cancer Patients

Gene Set Enrichment Analysis (GSEA) (v4.3.2) [10, 11] was used to measure enrichment of the "HP_BONE_PAIN" in a prostate cancer patient cohort from The Cancer Genome Atlas (TCGA)

(n = 554) (Project ID: TCGA-PRAD). Briefly, the Gene Cluster Text file (.gct) was generated from the TCGA prostate cancer patient dataset. To generate the Categorical Class file (.cls) patients were stratified into "high SCF" (n = 277) and "low SCF" (n = 277) expressing groups. Number of permutations for GSEA was set to 1,000 and for the Gene MatriX file (.gmx), the "HP_BONE_PAIN" gene list was used (HP:0002653). For the generation of the chip platform (.chip), the TCGA gene list was used. The GSEA platform determined significance and generated heat map data using Morpheus software developed by the Broad Institute.

Proteomics and Phosphoproteomics Analysis

Fresh DRG tissues were dissected from C57BL/6 mice as described in nerve sprouting assay methods and were incubated with NG medium for 30 min at 37°C, 5% CO_2. Subsequently, DRG were treated with either vehicle or 200 ng/mL SCF for 10 min or 1 hr. Following PBS wash, 200 µL RIPA Lysis Buffer (G-Biosciences, Cat. #:786490) supplemented with Protease Inhibitor Cocktail (APExBIO, Cat. #: K1007) and Phosphatase Inhibitor Cocktail (APExBIO, Cat. #: K1015) was added to each sample. DRG tissues were then homogenized by pestle on ice and placed on 4°C shaker for 1 hr followed by 20 min centrifugation at 16,000xg to remove debris. DRG lysates (200 µL) were further reduced and alkylated by adding 10 µL of 200 mM Tris(2-carboxyethyl)phosphine (TCEP) and incubation at 55°C for 1 hr, followed by addition of 10 µL of 375 mM iodoacetamide (IAM) and further incubation for 1 hr at room temperature. The protein pellet was isolated by overnight precipitation with 1 mL of chilled acetone at -20°C and centrifugation. The protein was reconstituted in 100 µL of 50 mM triethylammonium bicarbonate (TEAB). Following measurement of protein concentration by BCA, 100 µg protein was digested with 2.5 µg of sequencing grade modified trypsin overnight at 37°C followed by labeling of protein

fractions using the TMTpro 16-plex label reagent kit (Thermo Fisher Scientific, Cat. #: A44521) according to the manufacturer's protocol. All treatment groups included five biological replicates and the tandem mass tag for each sample was as follows: vehicle controls, 126, 127N, 127C, 128N, and 128C; 5-min SCF treatments, 129N, 129C, 130N, 130C, and 131N; 1-hr SCF treatments, 131C, 132N, 132C, 133N, and 133C. 120 μL from each sample was taken and pooled, while 5 μL was taken separately for quality control to assess labeling efficiency. The samples were acidified to pH 3 by thrifluoroacetic acid (TFAA) and purified using a 1cc C18 Sep-Pak cartridge. The sample was split into two fractions for i) phosphopeptide enrichment, and ii) high-pH reversed-phase (Hp-RP) peptide fractionation for global proteomics. Phosphopeptide enrichment was performed using a Fe-NTA Phosphopeptide Enrichment Kit (Thermo Fisher Scientific, Cat. #: A32992) according to the manufacturer's protocol. Hp-RP peptide fractionation was performed using a High pH Reversed-Phase Peptide Fractionation Kit (Thermo Fisher Scientific, Cat. #: 84868) according to the manufacturer’s protocol. Peptides were prepared in 5% acetonitrile with 1% formic acid for liquid chromatography-mass spectrometry (LC-MS/MS) analysis.

To perform LC-MS/MS analysis, samples were analyzed on an Orbitrap Eclipse Mass Spectrometer (Thermo Fisher Scientific) coupled with a Vanquish Neo nano-UHPLC system (Thermo Fisher Scientific) via the FAIMS (high-field asymmetric waveform ion mobility spectrometry) Pro interface. Peptides were separated on a DNV PepMap Neo (1500 bar, 75µm x 500mm) column for 120 min employing linear gradient elution consisted of water (A) and 80% acetonitrile (B) both of which contained 0.1% formic acid. Data were acquired using SPS (synchronous precursor selection)-MS3 based TMT method. MS2 scans were acquired for peptide identification using top-speed data-dependent scan where maximum number of MS2 spectra were

collected from fragmentation of selected precursor ions per 3 sec of cycle time between adjacent survey spectra (MS1). MS3 scan was sequentially performed for relative quantitation by multi-notch MS3-based TMT method where significant MS2 ions were selected by SPS with an assistance of real-time database search (RTS) which were fragmented to generate reporter ion peaks. The MS2-MS3 scan was repeated with precursor ion subsets isolated by FAIMS which compensation voltage was set to -35 eV, -45 eV, and -55 eV sequentially. Dynamic exclusion option was enabled which duration was set to 120 sec. To identify proteins, spectra were searched against the UniProt mouse protein FASTA database (17,082 annotated entries, Oct 2021) using the Sequest HT search engine with the Proteome Discoverer v2.5 (Thermo Fisher Scientific). Search parameters were as follows: FT-trap instrument; parent mass error tolerance, 10ppm; fragment mass error tolerance, 0.6 Da (monoisotopic); enzyme, trypsin (full); # maximum missed cleavages, 2; variable modifications, +15.995 Da (oxidation) on methionine, +304.207 Da (TMTpro) on lysine and N-term, +79.966 (phospho) on serine, threonine, and tyrosine; static modification, +57.021 Da (carbamidomethyl) on cysteine.

Ingenuity Pathway Analysis (IPA, QIAGEN) was performed to unveil the pathway behavior due to the differentially expressed and phosphorylated proteins using the data from phospho-proteomics analysis. For the analysis, the differential expression was defined, and phosphorylation was set at a cutoff of $p < 0.05$ compared to vehicle. The species and sample type were set as “Mouse” and “Tissues and Primary cells”, respectively. Based on the results of core analysis, the significantly enriched pathways ($p<0.01$) that are associated with neural physiological activities were ranked by z-score.

Single Cell RNA Sequencing and Analysis

A single cell suspension for single cell RNA sequencing (scRNA-seq) was performed according to the 10X Genomics guidelines. Briefly, ipsilateral lumbar DRGs (L2-L5) were collected from two sham-inoculated mice in a tube containing HBSS on ice. Samples were pooled in order to increase cell number for sequencing. The resulting DRGs were processed to single-cell suspensions. Cells with >80% viability were loaded into wells of a 10X Chromium single-cell capture chip targeting a cell recovery rate of 2,000-4,000 cells. Single-cell gel beads in emulsion were created on a Chromium Single-Cell Controller and scRNA-seq libraries were prepared using the Chromium Single-Cell 3' Library and Gel Bead kit according to the manufacturer's protocol (10X Genomics). Sequencing libraries were loaded at 1.3 PM on an Illumina NextSeq500 with High Output 150 cycle kit for paired-end sequencing using the following read length: 26 bp Read1, 8 bp i7 Index, 0 bp i5 Index, and 98 bp Read2. The Cell Ranger Single-Cell Software Suite v7.2 were used to perform sample de-multiplexing, alignment, filtering, and UMI (i.e., unique molecular identifier) counting. The data for each respective subpopulation were aggregated for direct comparison of single-cell transcriptomes. Low-quality cells identified as having less than 200 expressed genes were discarded. Cells with low viability were also removed if their proportions of mitochondrial gene expression are larger than 40%. t-SNE, K-means, and UMAP clustering were employed to reduce data dimensionality and to cluster cells based on global expression. Cell clusters were annotated based on cell type markers from prior studies [12].

Statistical Analyses

Numerical data are expressed as the mean ± standard error of the mean (SEM). Statistical analysis was performed using GraphPad Prism and SAS 9.4 statistical program (SAS Inc., Cary, NC) with

significance at $P \leq 0.05$. Outcome measures were transformed to satisfy the conditional normality assumption as needed. An unpaired *t* test or one-way analysis of variance (ANOVA) with Tukey's or Dunnett's post hoc test was used to compare single measurements between groups. For outcome measures (e.g., log-transformed radiance and adjusted guarding time) collected repeatedly over time, mixed-effects models were used to compare mean differences between groups over time. Group, time, and group-by-time interaction were included in the model. Animals were treated as a random effect. Contrasts were calculated to compare mean differences between groups at each time point.

1. Eber, M.R., et al., *Osteoblasts derived from mouse mandible enhance tumor growth of prostate cancer more than osteoblasts derived from long bone.* J Bone Oncol, 2021. **26**: p. 100346.

2. Schwei, M.J., et al., *Neurochemical and cellular reorganization of the spinal cord in a murine model of bone cancer pain.* J Neurosci, 1999. **19**(24): p. 10886-97.

3. Jimenez-Andrade, J.M., et al., *Preventive or late administration of anti-NGF therapy attenuates tumor-induced nerve sprouting, neuroma formation, and cancer pain.* Pain, 2011. **152**(11): p. 2564-74.
4. Peters, C.M., et al., *Endothelin and the tumorigenic component of bone cancer pain.* Neuroscience, 2004. **126**(4): p. 1043-52.
5. Chaplan, S.R., et al., *Quantitative assessment of tactile allodynia in the rat paw.* J Neurosci Methods, 1994. **53**(1): p. 55-63.
6. Peters, C.M. and J.C. Eisenach, *Contribution of the chemokine (C-C motif) ligand 2 (CCL2) to mechanical hypersensitivity after surgical incision in rats.* Anesthesiology, 2010. **112**(5): p. 1250-8.
7. Alamaw, E.D., et al., *Carprofen Attenuates Postoperative Mechanical and Thermal Hypersensitivity after Plantar Incision in Immunodeficient NSG Mice.* Comp Med, 2024. **74**(2): p. 105-114.
8. Eber, M.R., et al., *A Method of Bone-Metastatic Tumor Progression Assessment in Mice Using Longitudinal Radiography.* Methods Mol Biol, 2022. **2413**: p. 1-6.
9. Park, S.H., et al., *Usefulness of the measurement of neurite outgrowth of primary sensory neurons to study cancer-related painful complications.* Biochem Pharmacol, 2021. **188**: p. 114520.
10. Subramanian, A., et al., *Gene set enrichment analysis: a knowledge-based approach for interpreting genome-wide expression profiles.* Proc Natl Acad Sci U S A, 2005. **102**(43): p. 15545-50.
11. Mootha, V.K., et al., *PGC-1α-responsive genes involved in oxidative phosphorylation are coordinately downregulated in human diabetes.* Nature Genetics, 2003. **34**(3): p. 267-273.

12. Usoskin, D., et al., *Unbiased classification of sensory neuron types by large-scale single-cell RNA sequencing.* Nat Neurosci, 2015. **18**(1): p. 145-53.

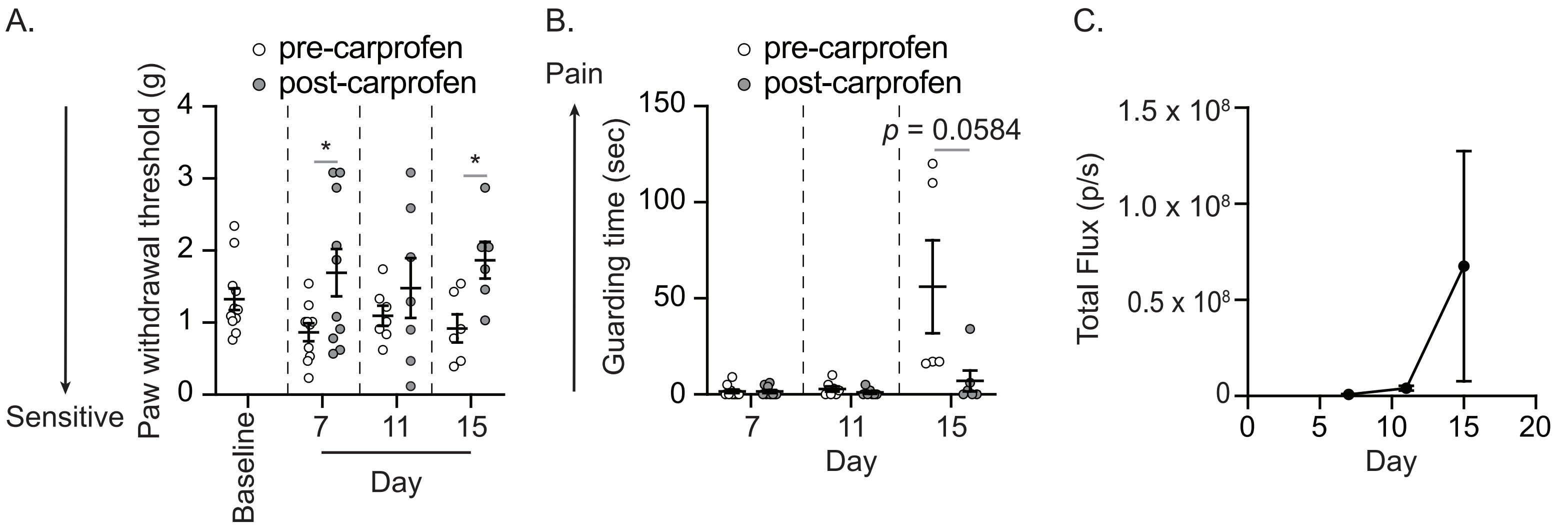
A.
pre-carprofen
post-carprofen
Sensitive
Paw withdrawal threshold (g)
4
3
2
1
0
*
*
Baseline
7
11
15
Day
B.
Pain
pre-carprofen
post-carprofen
Guarding time (sec)
150
100
50
0
p = 0.0584
7
11
15
Day
C.
Total Flux (p/s)
1.5 x 10^8
1.0 x 10^8
0.5 x 10^8
0
0
5
10
15
20
Day

Figure S1

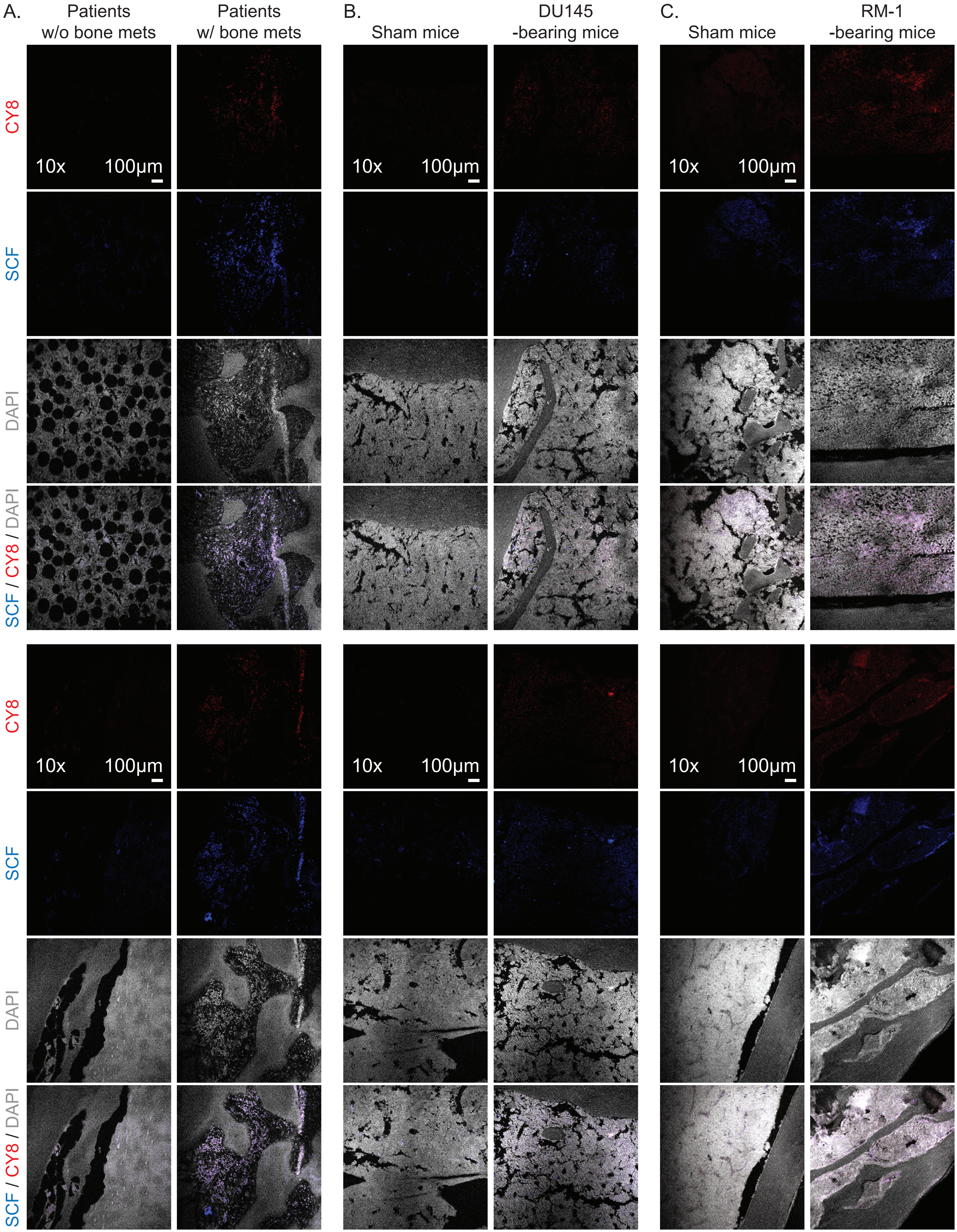
A.
Patients w/o bone mets
Patients w/ bone mets
B.
Sham mice
DU145 -bearing mice
C.
Sham mice
RM-1 -bearing mice
CY8
SCF
DAPI
SCF / CY8 / DAPI
10x
100µm

Figure S2

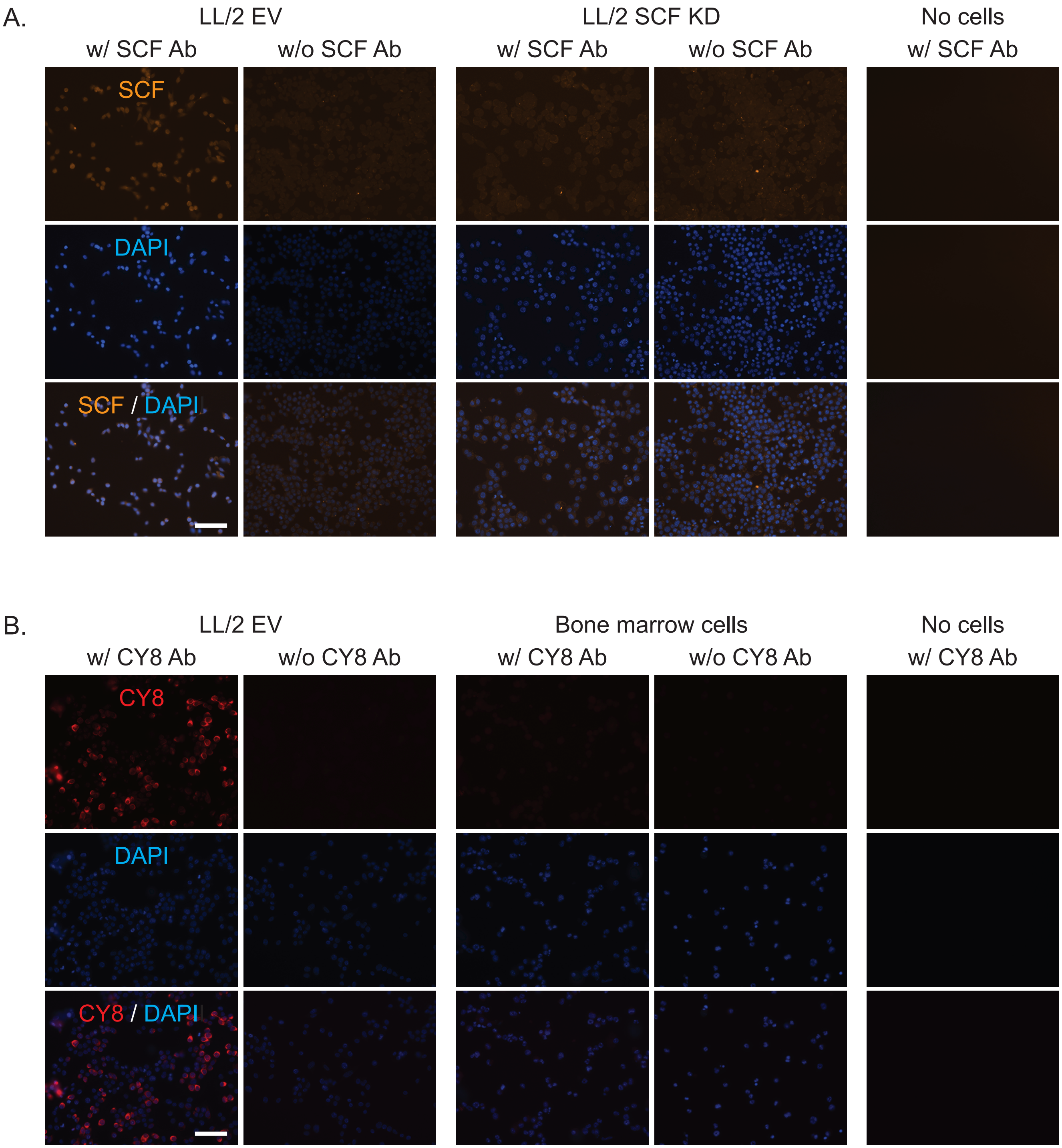
A.
LL/2 EV
LL/2 SCF KD
No cells
w/ SCF Ab
w/o SCF Ab
w/ SCF Ab
w/o SCF Ab
w/ SCF Ab
SCF
DAPI
SCF / DAPI
B.
LL/2 EV
Bone marrow cells
No cells
w/ CY8 Ab
w/o CY8 Ab
w/ CY8 Ab
w/o CY8 Ab
w/ CY8 Ab
CY8
DAPI
CY8 / DAPI

Figure S3
