# Supplementary material for: The contribution of stem cell factor and its receptor c-Kit to cancer-induced bone pain
Source: JCI Insight. 2026 May 5;11(12):e191905. doi: 10.1172/jci.insight.191905 (PMC13313531; doi:10.1172/jci.insight.191905)
Supplement: Unedited blot and gel images [file jciinsight-11-191905-s137.pdf]

A. Full unedited blot/gel for Figure 1E

Phospho c-Kit | c-Kit | GAPDH

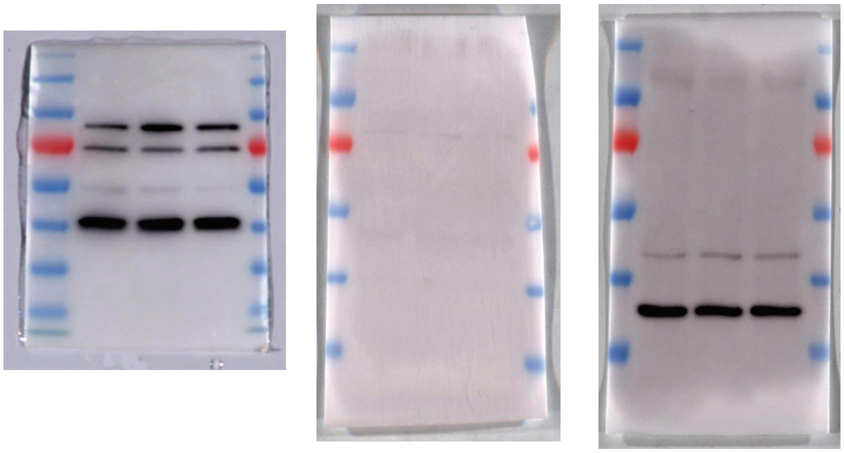

B. Full unedited blot/gel for Figure 9A

Phospho c-Kit | c-Kit | Phospho Akt | Akt | Phospho Erk | Erk

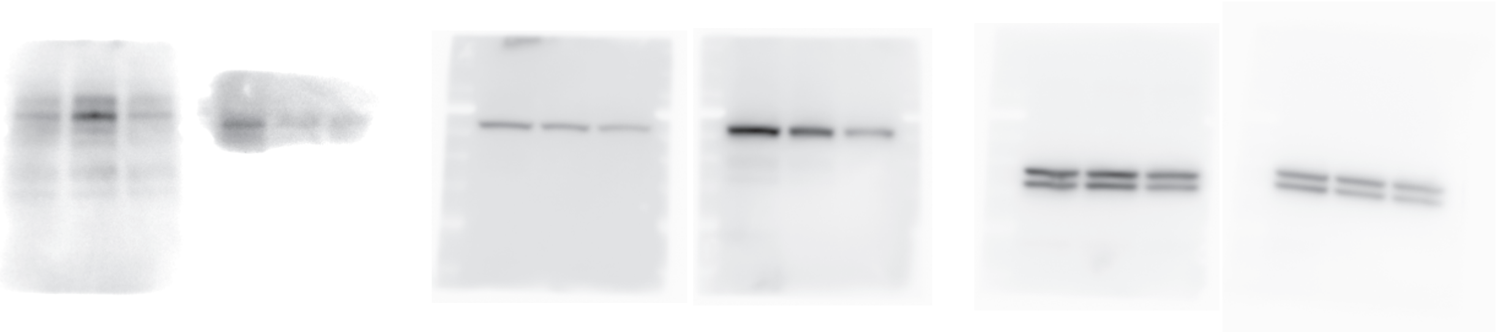

Phospho p38 | p38 | GAPDH

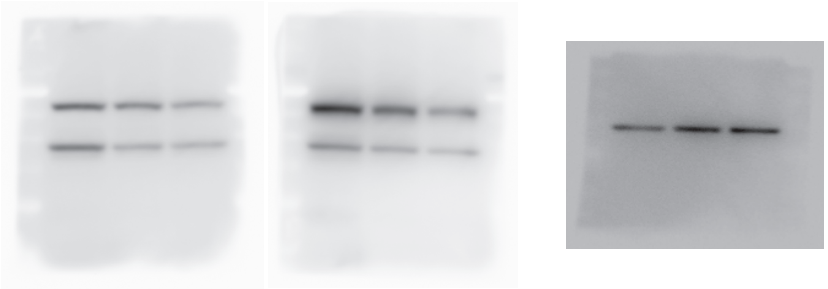

C. Full unedited blot/gel for Figure 9F

FGF1 | GAPDH

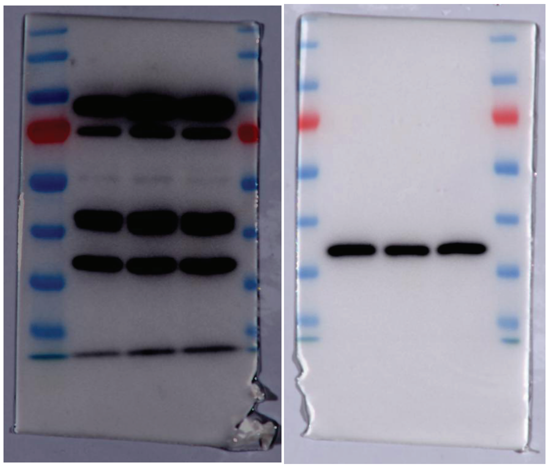

D. Full unedited blot/gel for Figure 9G

Phospho FGF1 | Ponceau

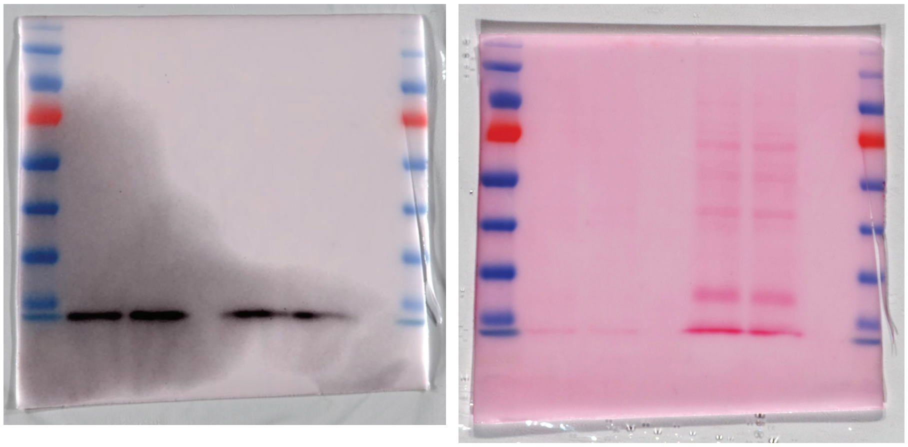

Full unedited blot/gel for Figures 1E, 9A, 9F, & 9G
